# Tripartite motif 25 ameliorates doxorubicin-induced cardiotoxicity by degrading p85α

**DOI:** 10.1038/s41419-022-05100-4

**Published:** 2022-07-23

**Authors:** Yihui Shen, Hui Zhang, Yangyue Ni, Xuejun Wang, Yifan Chen, Jiahui Chen, Yan Wang, Jinyi Lin, Yuchen Xu, Jian-Yuan Zhao, Leilei Cheng

**Affiliations:** 1 Department of Echocardiography, Zhongshan Hospital, Fudan University, Shanghai Institute of Cardiovascular Diseases, Shanghai Institute of Medical Imaging, 180 Fenglin Road, Shanghai, China; 2 Department of Cardiology, Zhongshan Hospital, Fudan University, Shanghai Institute of Cardiovascular Diseases, 180 Fenglin Road, Shanghai, China; 3 State Key Laboratory of Cell Biology, Center for Excellence in Molecular Cell Science, Shanghai Institute of Biochemistry and Cell Biology, Chinese Academy of Sciences, University of Chinese Academy of Sciences, Shanghai, China; 4 Department of Medical Oncology, Zhongshan Hospital, Fudan University, Shanghai, 200032 China; 5 Institute for Developmental and Regenerative Cardiovascular Medicine, MOE-Shanghai Key Laboratory of Children’s Environmental Health, Xinhua Hospital, Shanghai Jiao Tong University School of Medicine, Shanghai, 200092 China

**Keywords:** Proteomics, Heart failure

## Abstract

Doxorubicin (DOX)-based chemotherapy is widely used to treat malignant tumors; however, the cardiotoxicity induced by DOX restricts its clinical usage. A therapeutic dose of DOX can activate ubiquitin-proteasome system. However, whether and how ubiquitin-proteasome system brings out DOX-induced cardiotoxicity remains to be investigated. Here we conducted a proteomics analysis of a DOX-induced cardiotoxicity model to screen the potentially ubiquitination-related molecules. Dysregulated TRIM25 was found to contribute to the cardiotoxicity. In vivo and in vitro cardiotoxicity experiments revealed that TRIM25 ameliorated DOX-induced cardiotoxicity. Electron microscopy and endoplasmic reticulum stress markers revealed that TRIM25 mitigated endoplasmic reticulum stress and apoptosis in DOX-induced cardiomyocytes. Mechanistically, the Co-immunoprecipitation assays and CHX pulse-chase experiment determined that TRIM25 affected p85α stability and promoted its ubiquitination and degradation. This leads to increase of nuclear translocation of XBP-1s, which mitigates endoplasmic reticulum stress. These findings reveal that TRIM25 may have a therapeutic role for DOX-induced cardiotoxicity.

## Introduction

Anthracycline-based chemotherapy has been widely used to treat malignant tumors for the past eight decades. Unfortunately, based on previous studies, anthracyclines such as doxorubicin (DOX) can result in cardiotoxicity progressing to heart failure [1, 2]. A recent multicenter cohort study also showed that patients with cancer have a substantially increased risk of death caused by cardiovascular diseases even 40 years after initial chemotherapy treatment [3]. Several mechanisms may be responsible for DOX-induced cardiotoxicity, such as an excess of reactive oxygen species (ROS), mitochondrial dysfunction, and autophagy. However, optimal interventions for DOX-induced cardiotoxicity are yet to be fully determined [4, 5]; thus, further research is warranted to better understand the mechanism of DOX-induced cardiotoxicity.

Various studies have indicated that protein degradation by the ubiquitin-proteasome system regulates many fundamental biological processes in cardiovascular development and disease [6, 7]. Dysfunctional expression of ubiquitin E3 ligases, which mainly determines the specificity and rate-limiting step of ubiquitination, plays an essential role in the pathological processes of various diseases [8, 9]. Regulating the levels of ubiquitin E3 ligases has been proven to be able to treat many cardiovascular diseases, such as cardiac hypertrophy, valve calcification, and pulmonary arterial hypertension [10–12].

Recent studies have revealed that a therapeutic dose of DOX can activate ubiquitin-proteasome system-mediated proteolysis in cardiomyocytes [13]. The levels of some ubiquitin E3 ligases, such as atrogin-1, were increased in response to DOX treatment [14, 15]. Given that the expression patterns of ubiquitin E3 ligases in DOX-induced cardiotoxicity are yet to be elucidated, we conducted proteomics analysis of a DOX-induced cardiotoxicity model. Among the identified ubiquitin E3 ligases, TRIM25 is one of the tripartite motif-containing (TRIM) family proteins, which contain a RING finger domain followed by one or two B-Box domains and a coiled-coil domain [16]. Various studies have reported that TRIM family proteins play an indispensable role in regulating the antiviral innate immune response, metabolism, etc., via their ubiquitin ligase activity. Disorders of TRIM16 are reportedly involved in lipid metabolism in hepatocytes [17]. For example, overexpression of TRIM16 controls hepatic lipid accumulation and inflammation through the regulation of p-TAK1. Moreover, TRIM25 is essential for RIG-I ubiquitination, which is crucial for the RIG-I-mediated interferon-β production [18]. However, the role of TRIM25 in cardiovascular disease remains unknown.

Thus, we aimed to identify the expression patterns of ubiquitin E3 ligase in DOX-induced cardiotoxicity, and to investigate the role of TRIM25 in DOX-induced cardiotoxicity as well as its potential molecular mechanisms.

## Material and methods

### Mice

Animals were housed in a specific pathogen-free facility according to the guidelines of the Care and Use of Laboratory Animals (published by the national institutes of health, NIH publication no.86-23, revised 1996). The experiments were approved by the animal care and use committee of Zhongshan Hospital Research Ethics. Adult male C57BL/6 mice (8–10 weeks old, weighing 20–24 g), purchased from Shanghai Laboratory Animal Center of Fudan university (Shanghai, China) were randomly assigned to each group for studies on Dox-induced cardiotoxicity (*n* = 7–10). Sample size was determined by power calculations based on data from preliminary experiments with an α level of 0.05 and power of 0.80, a minimum number of 5 mice per group was required. A simple and free online randomization tool was performed by GraphPad (https://www.graphpad.com/quickcalcs/randomize1.cfm). After the randomized allocation of animals to the treatments, animals, samples, and treatments are coded and recorded until the data are analyzed.

### In vivo treatments

To generate DOX-induced cardiotoxicity, male C57/B6 mice were administered weekly intraperitoneal injections of DOX (5 mg/kg on days 0, 7, 14 and 21) for 4 weeks and heart function was assessed by echocardiography, 8 weeks after the first injection of DOX (cumulating dose 20 mg/kg of body weight). DOX was freshly dissolved in 0.9% NaCl (1 mL/kg). The saline group received an equal volume of fluid in the same period.

To investigate ER stress in vivo, mice were treated i.p. with a single injection of 5 mg/kg DOX. Mice were sacrificed and hearts collected for transmission electron microscopy analysis 4 weeks and 8 weeks after dox injection separately.

To determine the ability of anti-ER stress chemical agent TUDCA (Tauroursodeoxycholic Acid) to relieve DOX-induced cardiotoxicity in vivo, mice were treated i.p. with 300 mg/kg TUDCA or vehicle (DMSO) daily for 4 weeks, commencing on the one day before DOX treatment [19].

To examine the effect of TRIM25 on ER stress responses in vivo, mice were treated i.p. with 2 mg/kg TM (Tunicamycin), and 1 mg/kg TM (Tunicamycin) or vehicle (DMSO) 1 h before injection of DOX (on days 0, 7, 14 and 21). Given that the mice treated with 2 mg/kg TM (Tunicamycin) all died within 1 weeks, the mice treated with 1 mg/kg TM were used for later assessment.

Cardiomyocyte-restricted, genetic overexpressed of TRIM25 was performed by i.v. injection of 10 μl of AAV9-cTNT-GFP-TRIM25 (1 × 10^13^ v.g/ml; Genechem, shanghai, China) in 8-week-old C57/B6 mice (cumulating dose 1 × 10^11^ v.g per mouse). Similarly, to specifically overexpress p85α in the myocardium, 8-week-old C57/B6 mice received a single intravenous injection of 20 μl of AAV9-cTNT-GFP-p85α (0.5 × 10^13^ v.g/ml; Genechem, shanghai, China) (cumulating dose 1 × 10^11^ v.g per mouse). Dox was injected 4 weeks after the injection of AAV9.

### Echocardiography and measurements

Transthoracic echocardiography was performed using a 30 MHz high-frequency scan probe (VisualSonics Vevo2100; VisualSonics, Toronto, Canada) 8 weeks after the first injection of DOX. Anesthesia was induced by putting the mice in an induction chamber using 2% isoflurane (HEBEI YIPIN Pharmaceutical, Shanghai, China) and 2 L/min 100% oxygen for 1-2 min. Next, mice were placed on a constant temperature table in the supine position. M-mode images were recorded when the heart rate (HR) of the mice was maintained at 350–550 bpm. Heart rate (HR), left ventricular ejection fraction (LVEF), cardiac output (CO), fractional shortening (FS), left ventricular end-diastolic dimension (LVEDD), and left ventricular end-systolic dimension (LVESD) were measured as previously described [1]. All measurements were averaged for 3 consecutive cardiac cycles and were carried out by one experienced technician who was blinded to the experimental group identities.

### Isolation of neonatal mouse ventricular cardiomyocytes (NMVMs)

Neonatal mouse ventricular cardiomyocytes (NMVMs) were isolated and cultured as previously described [20]. Briefly, hearts of 1- to 3-day-old pups were dissected and atria were removed. Ventricles were minced and digested with repeatedly with 0.25% trypsin at 37 °C for 30 s to 1 min per cycle. When the cell supernatant became cloudy, the supernatant was neutralized by culture solution containing 10% serum. After 20–30 cycles of digestion, supernatant containing isolated cells were collected. The obtained cells were centrifuged 5 min at 800 rpm to separate non-myocardial cells and cultured at 37 °C in a humidified 5% CO2 incubator. One hour later, the supernatant containing suspended cardiomyocytes was transferred into other plates to reduce fibroblast contamination. Next, cell supernatant was passed through a 100 μm filter. Cardiomyocytes were cultured in Dulbecco’s modified Eagle’s medium (DMEM) High Glucose, supplemented with 10% Fetal Bovine Serum (FBS), and 5 mM penicillin/streptomycin (Gibco, Carlsbad, CA) for 2-3 days. Cardiomyocytes were used in experiments after the cells formed a confluent monolayer and contracted in synchrony.

### Cell cultures

The mouse cardiac muscle cell line HL-1 and rat cardiomyoblast cell line H9C2 were purchased from the Cell Bank of the Chinese Academy of Science (Shanghai, China) and were cultured in DMEM (Dulbecco’s Modified Eagle Medium) High Glucose (Gibco, Shanghai, China) supplemented with 10% FBS, 5 mM penicillin/streptomycin (Gibco, Shanghai, China). Human embryonic kidney cells 293 T (HEK-293T) was kindly provided by Doc. Zhiyuan Zhang (Dept. of General Surgery, Zhongshan Hospital, Fudan University, Shanghai, China) and cultured in DMEM High Glucose supplemented with 10%FBS, 5 mM penicillin/streptomycin. Cells were passaged at 70–80% confluence by dissociation from plates using Trypsin-EDTA. All cells were incubated under 5% CO2 at 37 °C.

To avoid the effect of high glucose on ER stress pathway, we used DMEM Low Glucose in DOX -induced ER stress model in vitro.

### Cell transfection and virus infection

To study the effect of TRIM25 on the HL-1 cells’ viability, HL-1 cells were infected with shrna -TRIM25, for 24 h using Lipofectamine^™^ 3000 Transfection Reagent (#L3000015, ThermoFisher, Shanghai, China) according to the manufacturer’s Reagent protocol.

Expression plasmids encoding p85α (encoded by Pik3r1), ubiquitin (Ub), TRIM25-WT, TRIM25-2EA, p85α-K506R, and p85α-K511R (p85α mutants with lys506 and lys511 changed into Arg were termed as K506R and K511R) were purchased from Genechem (Genechem, Shanghai, China). All plasmids were transfected with Lipofectamine^™^ 3000 Transfection Reagent (#L3000015, ThermoFisher, Shanghai, China) according to the protocal.

Adenovirus-driven ectopic expressing TRIM25 was achieved by infecting cultured cells at 50 MOI.

The reagents used and their commercial sources were indicated as follows: Doxorubicin (DOX) (selleckchem), cycloheximide (CHX) (selleckchem), MG132 (selleckchem), Tauroursodeoxycholic Acid (TUDCA) (selleckchem), Tunicamycin (TM) (MedChemExpress, Shanghai, China) and protein G agarose beads (#16–266, Merck Millipore, USA).

### Transmission electron microscopy analysis

Hearts samples were harvested from mice. 1 mm^3^ heart pieces from the left ventricular wall were fixed in 1.25% glutaraldhehyde (pH 7.2, Servicebio, Wuhan, China) overnight at 4 °C. Heart samples were washed in 0.1 M sodium cacodylate (3 times for 30 min), post-fixed and thin sections were imaged on a HITACHI electron microscopy (HT 7800, Japan).

### Quantitative real-time PCR

Total RNA extraction of the cells or heart tissues was extracted using RNAiso Plus (#9109, TAKARA, China) and 1 μg RNA was reverse transcribed by the PrimeScript^™^ RT Master Mix (Perfect Real Time) (# RR036A, TAKARA, China). Quantitative real-time (qRT) PCR was performed by SYBR Green PCR Master Mix (Applied Biosystems, CA) in the ABI 7300 Detection System (Applied Biosystems, CA). The qRT-PCR primers sequences of the detected genes were showed in the Supplementary Table 2.

### Western blot analysis

Total proteins isolated from the left ventricle of heart tissues, NMVMs, HL-1, H9C2, and HEK-293T cells were lysated in ice-cold lysis buffer for 30 min with protease and phosphatase inhibitors (Thermo Fisher Scientific, MA, USA). The protein concentration was quantified using BCA protein assay kit (Pierce^™^ BCA Protein Assay Kit, Thermo Scientific, China) following the manufacturer’s instructions. Protein (30 μg/lane) was loaded on SDS-PAGE and then transferred to 0.22μm PVDF membranes (Merck Millipore, Billerica, MA, USA). The membranes were blocked with 5% BSA (Bovine serum albumin, Sigma, USA) for 2 h at room temperature and incubated at 4 °C overnight with primary antibodies. The primary antibodies used in this study are listed in Supplementary Table 1. After washing with TBST (Sangon Biotech, Shanghai, China), the membranes were incubated with a horseradish peroxidase HRP-conjugated antibody #7076 S、#7074 S(1:3000, Cell Signaling Technology, USA). GAPDH or Lamin A/C was used as an internal control. The proteins were visualized using an ECL Western blotting detection system #WBKLS0500 (MILLIPORE, USA). The density of each band was quantified by densitometric analysis with ImageJ software (Image Processing and Analysis in Java, National Institutes of Health, USA).

### Morphological and histological analysis

The hearts were perfused with PBS (Phosphate Buffered Saline, Hyclone) and then isolated to measure their weight. The heart samples were fixed with 4% paraformaldehyde and embedded in paraffin. The paraffin-embedded hearts were sectioned at a thickness of 5 μm and stained with eosin (H&E) and Masson’s trichrome for the analysis of atrophy and fibrosis according to the manufacturer’s recommended protocol (Servicebio Biotech, Wuhan, China). For measurements, 5 random high-power fields from 5 sections of each heart were chosen and quantified in a blinded manner. The cross-sectional area (CSA) of the cardiomyocytes and the area of the cardiac fibrosis were evaluated through morphometric analysis of the H&E-stained and Masson’s trichrome sections respectively. Specimens were observed under a light microscope (400 amplification; Nikon Tokyo, Japan). The images were measured through an automated image analysis system (Image-Pro Plus 5.0).

### Tissue preparation and immunofluorescence analysis

For IF assay, the 5 μm cryosections were fixed in 4% paraformaldehyde for 30 min and next washed in phosphate buffered saline (PBS) for 3 times. The sections were permeabilized with 0.3% Triton X-100 in PBS for 10 min and blocked with 10% normal goat serum (Beyotime, Jiangsu, China) for 1 h at RT. The slides were then incubated with primary antibodies overnight at 4 °C, followed by secondary antibodies at RT for 1 h. The images were captured using fluorescence microscope (Nikon Tokyo, Japan) or Zeiss Pascal confocal microscope (Zeiss, FV3000, Germany), quantified by ImageJ software (https://imagej.nih.gov/ij/).

### Evaluation of apoptosis

TUNEL labeling was conducted in accordance with the manufacturer’s protocol (One Step TUNEL Apoptosis Assay Kit; Beyotime, Jiangsu, China). In brief, the paraffin-embedded slides were incubated with 50 μL of TUNEL reaction mixture containing terminal deoxynucleotidyl transferase (TdT) and biotinylated dUTP for 1 h at 37°C. After washing with PBS three times, Hoechst 33258 was dispensed dropwise onto the slides and incubated for 5 min at the room temperature. The apoptosis-positive cells were counted in 6 randomly selected fields from each slide. The results were recorded as the ratio of TUNEL-positive nuclei/Hoechst 33258-stained nuclei and analyzed via fluorescence microscopy.

### Proteomics analysis

The samples from PBS and DOX treated HL-1 cells were added with appropriate amount of SDT cracking solution, boiling water bath for 15 min. 14000 *g* centrifuged for 15 min, the supernatant was removed. Carryon protein quantitative analysis by BCA method. Each sample was labelled with 100 μg Peptide, according to TMT 6/10plex Isobaric Mass Tag Labeling kit (Thermo Fisher scientific). After that, labeled samples were separated by Easy nLC system (Thermo Fisher Scientific, Waltham, MA, USA) and processed for gradient elution by a analytical column (Thermo Fisher Scientific, Acclaim PepMap RSLC 50 um X 15 cm). The samples were analyzed for TMT Quantitative proteomics by Q Exactive plus Mass Spectrometer. The raw data of mass spectrometry analysis was conducted inventory identification and quantitative analysis using software Mascot 2.6 and Proteome Discoverer2.1. Proteins with a 1.2-fold change (≥1.2 or ≤0.83) and correspongding adjusted *P* < 0.05 were identified as target molecules and entered into the Database for Annotation, Visualization and Integrated Discovery (DAVID, http://david.abcc.ncifcrf.gov) and then subjected to Gene Ontology (GO) and Kyoto Encyclopedia of Genes and Genomes (KEGG) pathway database. The proteomics analysis was performed and analyzed by Genechem (Shanghai).

### Bioinformatics analysis

We analyzed a GEO RNA-seq dataset (GSE40289) generated from the cardiomyocytes with or without DOX treatment from mice isolated by using a Langendorff apparatus, with the tool R language (Version 3.61) used to analyse the raw data. The lists of differentially changed ubiquitin E3 ligase in cardiomyocytes treated with DOX were shown in Supplementary Table 3. Spectrum of total ubiquitin E3 ligases were from Integrated Genomic Analysis [21].

### Identify TRIM25 and p85α interaction proteins by mass spectrometry

Five 10 cm dishes HL-1/HEK293T cells were transfected with empty vectors or GV314-3Flag-p85α or GV314-3Flag-TRIM25 with Lipofectamine 3000 and continued cultured for 48 h. Then cells were lysed in Co-IP buffer (50.0 mM Tris–HCl, 150.0 mM NaCl, 5.0 mM EDTA, and 1.0% NP-40 pH 7.6) supplemented with protease inhibitor cocktail (HY-K0010, MedChemExpress, China), and incubated with anti-Flag affinity gels (A4596, Sigma-Aldrich, USA) overnight at 4 °C. The immunoprecipitates were washed three times with COIP buffer. Bound proteins were analysed by immunoprecipitation-mass spectrometry.

### Co-immunoprecipitation

For co-immunoprecipitation, assays were performed as described previously [22]. Briefly, tissues/cells were homogenized in COIP buffer (50 mM Tris–HCl, 150 mM NaCl, 5 mM EDTA and 1% NP-40 pH 7.6) supplemented with protease inhibitor cocktail (MedChemExpress, Shanghai, China). Then, cell lysates were incubated and rocked with indicated antibody and Protein G agarose beads (16-266, Millipore, Shanghai, China), or affinity gels (Sigma-Aldrich, Shanghai, China) overnight at 4 °C. Proteins bound to protein G-Sepharose were washed, and denatured in 2× SDS-PAGE loading buffer at 100 °C for 10 min. Then proteins were subjected to SDS-PAGE and analyzed by Western Blot.

### Expression and purification of recombinant proteins

Expression and purification of recombinant proteins were performed as described previously [23]. Briefly, GST- or His6 (His)-tag proteins were expressed in the BL21 (DE3) *Escherichia coli* cells. After IPTG (isopropyl β-D-thiogalactoside, Sigma, USA) induction, cells were pelleted, lysed and incubated with glutathione or Ni2^+^ IDA resin to enrich the respective proteins, eluted with 40 mM reduced lutathione (GSH, Sigma) or 100 mM imidazole dissolved in Tris buffer (pH 8.0). Protein Expressed as inclusion bodies was dialyzed in PBS buffer supplemented with 4 mM GSH, 0.4 mM GSSG, 0.4 M L-Arginine, 1 M Urea. The purity of recombinant protein was examined by SDS-PAGE with Coomassie blue staining and Western Blot, then aliquoted and preserved in 20% glycerol at −80 °C.

### GST pull-down assay

The pull-down assay was conducted as by the instruction (Pierce^TM^ GST Protein Interaction Pull-Down Kit, #21516, Thermo scientific, USA). Briefly, the purified GST- p85α or GST-empty (Bait Protein, approximatedly 150 ug of bait protein) were added to the Pierce spin Column and incubated at 4 °C for 30 min with gentle rocking motion on a rotating plaform. Next, His-TRIM25 (Prey Protein) was incubated with the GST-tagged bait protein at 4 °C for 1 h. Then the beads were washed five times with washing buffer. Next, bait-prey protein was eluted and then centrifuged at 1250 *g* for 30 s. The bait-prey protein was denatured at 100 °C for 10 min in 2x SDS-PAGE loading buffer and subjected to Coomassie blue staining or western blot analysis.

### Statistical analysis

All data are expressed as the mean±standard error of the mean (SEM). Comparison between two groups was performed by the two-tailed Student’s *t*-test. Comparisons between multiple groups were conducted using a one-way ANOVA analysis with Bonferroni’s multiple conparisons test. Before using statistical analysis, Homogeneity of variance test was tested by bartlett testing. All data analysis was conducted by Prism 8.0 (GraphPad Software, CA, USA). A value of *P* < 0.05 was considered as statistically significant. The precise *P*-value of significant changes were shown in each figure.

## Results

### Expression patterns of ubiquitin E3 ligases in doxorubicin-induced cardiotoxicity

**Fig. 1:**
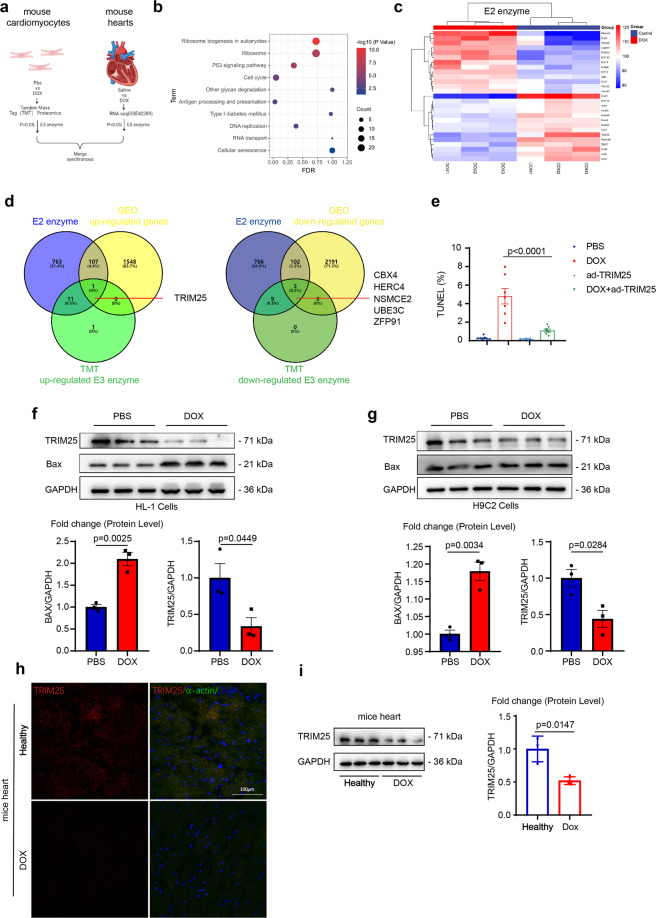
Ubiquitin E3 ligase TRIM25 level is decreased in DOX-treated cardiomyocytes. **a** Scheme showing the procedure of identifying ubiquitination-associated targets in DOX-induced cardiotoxicity. **b** Kyoto Encyclopedia of Genes and Genomes pathway mapping of differentially expressed proteins of DOX-treated cardiomyocytes and control cardiomyocytes. **c** Hierarchical clustering heatmap of the profile of differentially expressed genes from cardiomyocytes between DOX and phosphate‐buffered saline hearts. **d** Venn diagram showing differentially expressed ubiquitin-related targets consistent in cell and tissue models. **e** Percentage of TUNEL-positive nuclei in neonatal mouse ventricular myocytes (NMVMs) of each experimental group. **f, g** Western blot analysis of TRIM25 and Bax in HL-1 and H9C2 cells treated by DOX for 6 h. **h** Localization and quantification of TRIM25 (red) in heart sections from mice treated by DOX for 6 h by confocal laser scanning immunofluorescence. The nucleus is labeled by DAPI (blue). **i** Western blot analysis of TRIM25 levels from the hearts of mice treated by DOX for 6 h. Experiments were independently repeated three times. Data are represented as the means ± SEM. *P* values were determined by using one-way ANOVA with Bonferroni *post hoc* test (**d**) and unpaired two-tailed Student’s *t* test (*n* = 4, C) (*n* = 3, J). Scale bar, 100 μm in I. DOX, doxorubicin; CON, control; NS not significant.

To investigate the expression profile of ubiquitin E3 ligases in cardiomyocytes in response to DOX, we performed tandem mass tag proteomics of a DOX-induced cardiotoxicity cell-based model, which consisted of HL-1 mouse cardiomyocytes treated with DOX for 16 h. Furthermore, we searched an RNA-seq (GSE40289) of a tissue-based model to identify stable ubiquitination-associated candidate molecules (Fig. 1a). A total of 6326 proteins were detected in the proteomics results, and 1858 proteins were differentially expressed between DOX-treated and normal cardiomyocytes. Among these, 231 were significantly upregulated in DOX-treated cardiomyocytes whereas 167 were downregulated (fold change >1.2 or fold change <0.83; *P* < 0.05). Using the Kyoto Encyclopedia of Genes and Genomes as well as gene ontology analysis, we found that DOX treatment activated the signaling pathways related with P53 signaling and the cell cycle, indicating the successful inducement of cardiotoxicity (Fig. 1b and Supplementary Fig. 1). Subsequently, differentially expressed E3 ligases identified by proteomics analysis are listed in Fig. 1c, whereas differentially expressed E3 ligases based on the RNA-seq (GSE40289) analysis are listed in Supplementary Table 3. Regarding the identification of ubiquitination-associated candidate targets, which were consistent in both cell and tissue models, we found that five E3 ligases met the screening criteria, namely TRIM25, CBX4, HERC4, NSMCE2, UBE3C, and ZFP91, among which TRIM25 showed the greatest expression alteration (Fig. 1d and Supplementary Fig. 2).

To explore the role of TRIM25 in the model of DOX-induced cardiotoxicity, primary cultured mouse cardiomyocytes as a well-validated cardiomyocyte model were infected with TRIM25-overexpressing adenovirus (Ad-TRIM25) (MOI 50), confirmed by fluorescent staining, and then treated with DOX (Supplementary Fig. 3). Consistent with previous studies [24, 25], DOX treatment induced substantial apoptosis, as indicated by the increased percentage of terminal dUTP nick-end labeling (TUNEL) positive cells compared with phosphate‐buffered saline treatment. Besides, apoptosis-related proteins were also upregulated after DOX treatment. Western blotting confirmed that the levels of pro-apoptosis proteins, such as Bax and cleaved caspase-3, were increased by DOX treatment, whereas that of the anti-apoptosis protein Bcl2 was decreased. Surprisingly, Ad-TRIM25 treatment significantly ameliorated DOX-induced apoptosis (Fig. 1e and Supplementary Fig. 4a, b, c).

Next, we assessed the expression of TRIM25 after treatment with DOX in vitro in a time-dependent manner by western blotting. The early expression of TRIM25 was markedly decreased with a significant increase in Bax levels. Contrastingly, when TRIM25 expression started to increase after 16 h, Bax levels did not increase synchronously (Supplementary Fig. 5). We hypothesized that low TRIM25 expression induced by DOX in the acute phase could have contributed to the cardiotoxicity. TRIM25 expression levels were decreased in both mouse cardiomyocyte HL-1 cells and rat cardiomyocyte H9C2 cells in the acute phase, compared with healthy cells (Figs. 1f, g); this was also verified in heart tissues obtained from mice corresponding to a DOX-induced acute model of cardiotoxicity (Figs. 1f, g).

### TRIM25 is an E3 ligase of p85α

Given that TRIM25 is a ubiquitin E3 ligase, we explored the underlying mechanism of the cardioprotective role of TRIM25 overexpression via immunoprecipitation-mass spectrometry (IP-MS) analysis to identify the specific target. IP-MS demonstrated that the peptide fragment IFEEQCQTQERYSKEYIEK (Supplementary Fig. 6) belonged to p85α and had ubiquitin modified amino acid residues Gly-Gly in K14 and K19 after transfection with plasmid- Flag-tag-TRIM25 in HL-1 cells, indicating that TRIM25 maybe a ubiquitin E3 ligase for p85α to promote its ubiquitination and degradation. Furthermore, we also transfected HEK293T cells with plasmid-Flag-p85α, and the peptide fragment QAGLEAAAK, which belonged to TRIM25, was detected by IP-MS. Co-immunoprecipitation (CO-IP) assays also confirmed this interaction (Supplementary Fig. 7). In light of negatively correlated protein levels between TRIM25 and p85α in DOX-induced cardiotoxicity cell-based acute model (Supplementary Fig. 8), we postulated that TRIM25 diminished the DOX-induced cardiotoxicity via the regulation of p85α.

**Fig. 2:**
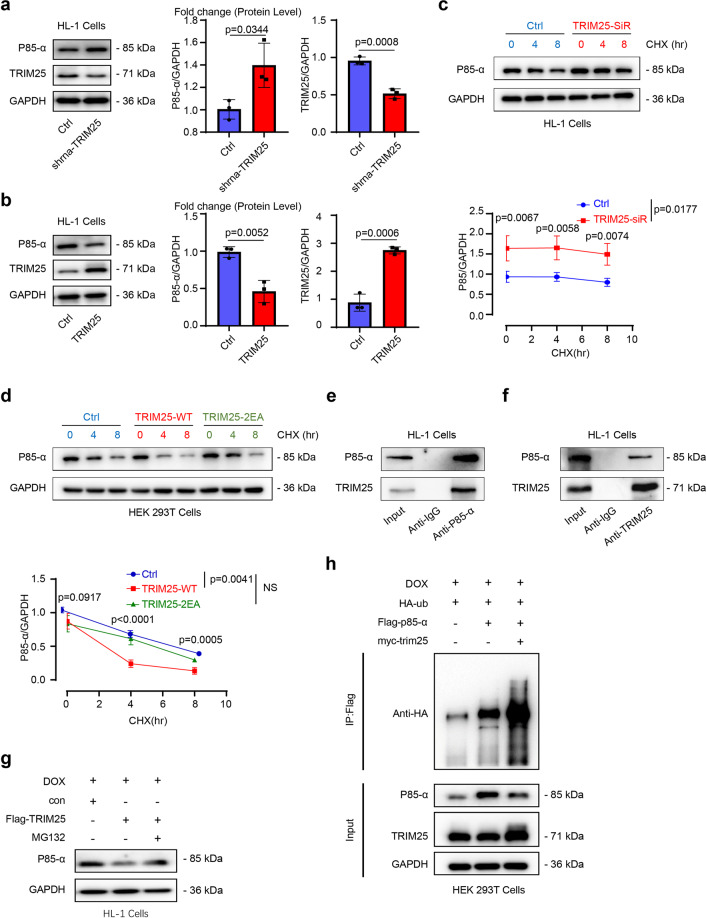
TRIM25 decreases p85α stability via ubiquitination. **a** Western blot analysis of p85α and TRIM25 levels in HL-1 cells transfected with shRNA-TRIM25 (1 μg/ml) or control shRNA. **b** Western blot analysis of p85α and TRIM25 in HL-1 cells transfected with TRIM25 overexpression plasmids (TRIM25-WT) (1 μg/ml) or control plasmid. **c** Representative immunoblot and relative quantification of p85α in HL-1 transfected with shRNA-TRIM25 or shRNA-Ctrl (1 μg/ml), then treated with CHX (100 μg/ml) for up to 8 h. **d** Representative immunoblot and relative quantification of p85α in HL-1 transfected with control plasmid, TRIM25-WT or TRIM25-2EA mutation plasmids (TRIM25-2EA) (1 μg/ml), then treated with CHX (100 μg/ml) for up to 8 h. **e, f** Co-immunoprecipitation (CO-IP) assay analyzed the interaction of endogenous TRIM25 and p85α in HL-1 cells. **g** Western blotting assessed the level of p85α in HL-1 cells treated with or without MG132 (20 μM) for 12 h. **h** HL-1 cells transfected with the plasmids as indicated and treated with DOX (1 μM) for 12 h. Immunoblot analysis of the cell lysates and p85α-IP with the indicated antibodies. Data represent the means ± SEM. (*n* = 3). P values were determined by unpaired 2-tailed Student *t* test (**a**, **b**) and two-way ANOVA between multiple groups (**c**, **d**). WT, wild-type; ctrl, control; TRIM25-2EA, TRIM25 mutant with Glu9 and Glu10 changed into Ala.

Next, we further explored whether TRIM25-regulated p85α stability and protein level via the ubiquitination of p85α. TRIM25 knockdown by shRNA increased p85α expression in HL-1 cells (Fig. 2a). Contrastingly, TRIM25 overexpression in both HL-1 and HEK293T cells decreased p85α expression levels (Fig. 2b and Supplementary Fig. 9). Moreover, we found that TRIM25 overexpression leads to a decrease in p85α degradation, but that TRIM25 knockdown prolonged p85α half-life in HEK293T cells (Fig. 2c). Furthermore, overexpression of the loss-of-function mutant TRIM25-2EA (TRIM25 mutant with Glu9 and Glu10 changed to Ala) did not affect the half-life of p85α (Fig. 2d). CO-IP assays showed that TRIM25 interacts with p85α endogenously in HL-1 cells (Figs. 2e, f). Moreover, reduced p85α levels can be rescued by the proteasome inhibitor MG132 (Fig. 2g). P85α overexpression induced by TRIM25 downregulation could be attenuated by TRIM25 overexpression, indicating that TRIM25 overexpression may induce p85α degradation (Fig. 2h). Taken together, these results indicated that TRIM25 ubiquitinated p85α and led to its degradation.

**Fig. 3:**
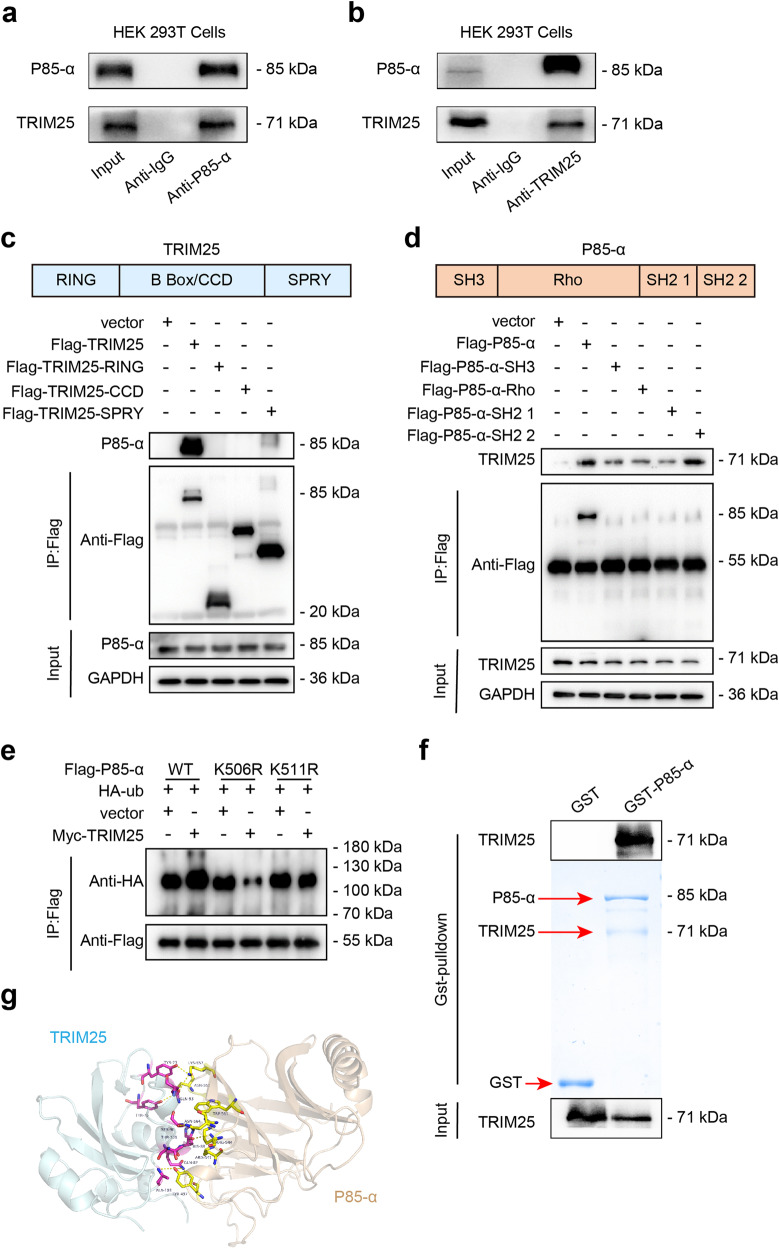
TRIM25 interacts and ubiquitinates p85α. **a, b** CO-IP assay of TRIM25 and p85α were performed to investigate the interactions between TRIM25 and p85α in HEK293 cells. **c** HEK293 cells overexpressing full-length and TRIM25 truncates (The RING zinc-finger (R), B-Box/coiled-coil (CC) and SPRY domain of TRIM25) were immunoprecipitated with the indicated antibody. **d** HEK293 cells overexpressing full-length or truncated p85α were immunoprecipitated with the indicated antibody. **e** Immunoblot analysis of the ubiquitination of wide-type and p85α mutations (K506R and K511R) with the indicated antibodies. **f** TRIM25 directly binds to p85α. Glutathione S-transferase (GST)–fused p85α and His-fused TRIM25 recombinant proteins were co-incubated and subjected to GST pulldown. **g** Computational molecular docking simulation analyses of TRIM25 to p85α.

Furthermore, the interaction between endogenous TRIM25 and p85α was validated in HEK293T cells (Figs. 3a, b). Next, we generated a series of truncated mutants of TRIM25 and p85α to detect functional domains of the TRIM25-p85α interaction. Similar to other TRIM family members, TRIM25 contains a cluster of RING, coiled-coil, and PRY/SPRY domains, all of which were constructed in our study. Binding analysis revealed that the PRY/SPRY domain of TRIM25 was bound to p85α as full-length TRIM25, whereas the RING and CC domains were not (Fig. 3c). Moreover, we found that the SH2 domain of p85α was crucial for its interaction with TRIM25 (Fig. 3d). Finally, IP-MS demonstrated that the peptide fragment IFEEQCQTQERYSKEYIEK (Supplementary Fig. 6) belonged to p85α and had ubiquitin modified residues amino acids Gly-Gly in K14 and K19 after transfecting HL-1 cells with Flag-TRIM25, suggesting that TRIM25 might target K506 and K511 of p85α to promote its ubiquitination and degradation. To verify this hypothesis, we constructed two mutants with Lys506 and Lys511 of p85α changed into Arg (termed as K506R and K511R). The biochemistry data showed that suppression of the ubiquitination level of p85α was observed when the p85α-K506R or K511R mutants were expressed, indicating that TRIM25 targets K506 and K511 of p85α to promote ubiquitination and degradation (Fig. 3e). Collectively, these data demonstrate that TRIM25 is an E3 ligase of p85α and can promote its degradation by enhancing its ubiquitination.

Glutathione S-transferase pulldown assays showed that p85α could interact with TRIM25 (Fig. 3f). Computational molecular docking simulation analyses of the PRY/SPRY domain of TRIM25 to the SH2 domain of p85α. Eight hydrogen bonding forces revealed the high possibility of combining structures (Fig. 3g).

### TRIM25 inhibits doxorubicin-induced cardiotoxicity

**Fig. 4:**
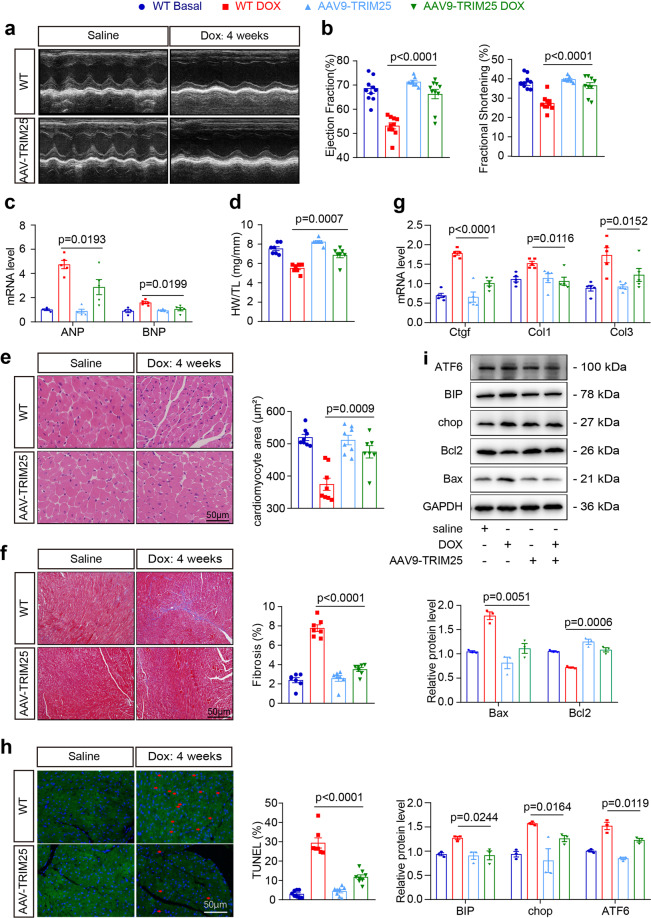
TRIM25 overpression by adeno-associated virus alleviates DOX-induced cardiotoxicity in mice. WT and TRIM25 overpression (OE) mice were injected by adeno-associated virus 9 via the tail vein, and after 4 weeks, they were treated with a cumulative dose of 20 mg/kg DOX or saline by 4 weekly injections (5 mg/kg on days 0, 7, 14 and 21). **a** Representative M-mode echocardiographic images of WT and AAV-TRIM25 hearts 8 weeks after the first DOX injection. **b** M-mode images from three consecutive cardiac cycles for each animal were used to calculate ventricular measurements: ejection fraction, fractional shortening. **c** Relative quantification of cardiac Anp (atrial natriuretic peptide) and Bnp (brain natriuretic peptide) mRNA expression levels. **d** The HW/TL (heart weight/tibia length) ratio as a cardiac atrophy index of DOX-treated WT and AAV-TRIM25 mice. **e** Representative images of H&E staining and relative quantification of cardiomyocyte area in heart sections from mice treated as in (**a**). **f, g** Representative images of Masson staining and relative quantification of collagen deposition in heart sections from mice. **h** Representative images of TUNEL staining (left) and quantification of TUNEL-positive nuclei per field (right) in heart sections from WT and AAV-TRIM25 mice treated with DOX or saline. **i** Immunoblot (upper) and relative quantification (lower) of indicated targets in whole hearts from WT and AAV-TRIM25 mice treated as in (**a**) (*n* = 3). *N* = 7–10 animals/group; Values represent mean ± SEM. *P* values were analyzed by one-way ANOVA test with Bonferroni *post hoc* test (**b**–**i**). Scale bar, 50 μm in **e**, **f** and **h**.

To explore the function of TRIM25 in DOX-induced cardiotoxicity, we used a chronic model by injecting adeno-associated virus 9 (AAV9) expressing TRIM25 (AAV9-TRIM25) into 8-week-old mice via the tail vein. The induction of cardiotoxicity was maintained by a four-week low-dose DOX treatment to simulate the course of clinical chemotherapy (Supplementary Fig. 10a). Cardiomyocyte-specific TRIM25 overexpression (OE) mice were established. Western blotting and quantitative polymerase chain reaction analyses confirmed the efficiency of TRIM25 overexpression in the heart (Supplementary Fig. 10b, c). After DOX administration, no significant differences were observed in body weight between the TRIM25 OE and DOX groups (Supplementary Fig. 11). Echocardiographic assessment revealed a significant diminishment of cardiac contractility and significant upregulation of myocardial atrial natriuretic peptide (Anp) and brain natriuretic peptide (Bnp) mRNA levels in wild-type (WT) mice treated by DOX (Fig. 4a, b, c and Supplementary Table 4). Conversely, the deterioration of left ventricular systolic function as well as the increase of Anp and Bnp mRNA levels were all mitigated in DOX-treated TRIM25 OE mice, demonstrating the protective effects of TRIM25 against DOX-induced cardiotoxicity.

Wasting of cardiomyocytes, fibrosis and apoptosis are acknowledged as key hallmarks in DOX-related cardiotoxicity. DOX-treated TRIM25 OE mice had normal cardiac mass and cardiomyocyte size compared with the WT DOX group (Fig. 4d, e). We found that fibrosis was mitigated in TRIM25 OE mice, as indicated by Masson staining, and that the expression of profibrotic genes in the heart was downregulated (Fig. 4f, g). Moreover, TUNEL staining and the expression of apoptosis proteins, such as Bax and Bcl2, revealed that apoptosis was significantly lower in AAV-TRIM25-DOX than in WT-DOX hearts (Fig. 4h, i).

Given that TRIM25 functions as a feedback mechanism that responds to endoplasmic reticulum (ER) stress, and that the dysregulation of ER homeostasis in the heart is implicated in cardiovascular disease, including DOX cardiomyopathy, we further examined the ER stress-related protein in heart tissues. The expression of BIP/GRP78, a master regulator for ER stress that activates unfolded protein response (UPR) signaling, was increased after treatment with DOX. Activating transcription factor 6 (ATF6), a type II transmembrane protein containing a basic leucine zipper motif, became activated to allow its interaction with ER stress elements and the regulation of the expression of genes encoding BIP and its components. C/EBP homologous protein (CHOP, also known as DDIT3) is a pro-apoptotic mediator. The overexpression of ATF6 and CHOP were downregulated by TRIM25 overexpression (Fig. 4i).

Overall, these results demonstrated that TRIM25 overexpression protects against DOX-induced upregulation of pathways and genes promoting apoptosis, fibrosis, and ER stress.

### TRIM25 mitigates doxorubicin-induced ER stress in cardiomyocytes

To investigate the mechanisms associated with TRIM25-induced cardioprotection, we performed in vitro analyses of ER stress, which is considered to be a potential contributor to DOX-induced cardiotoxicity.

**Fig. 5:**
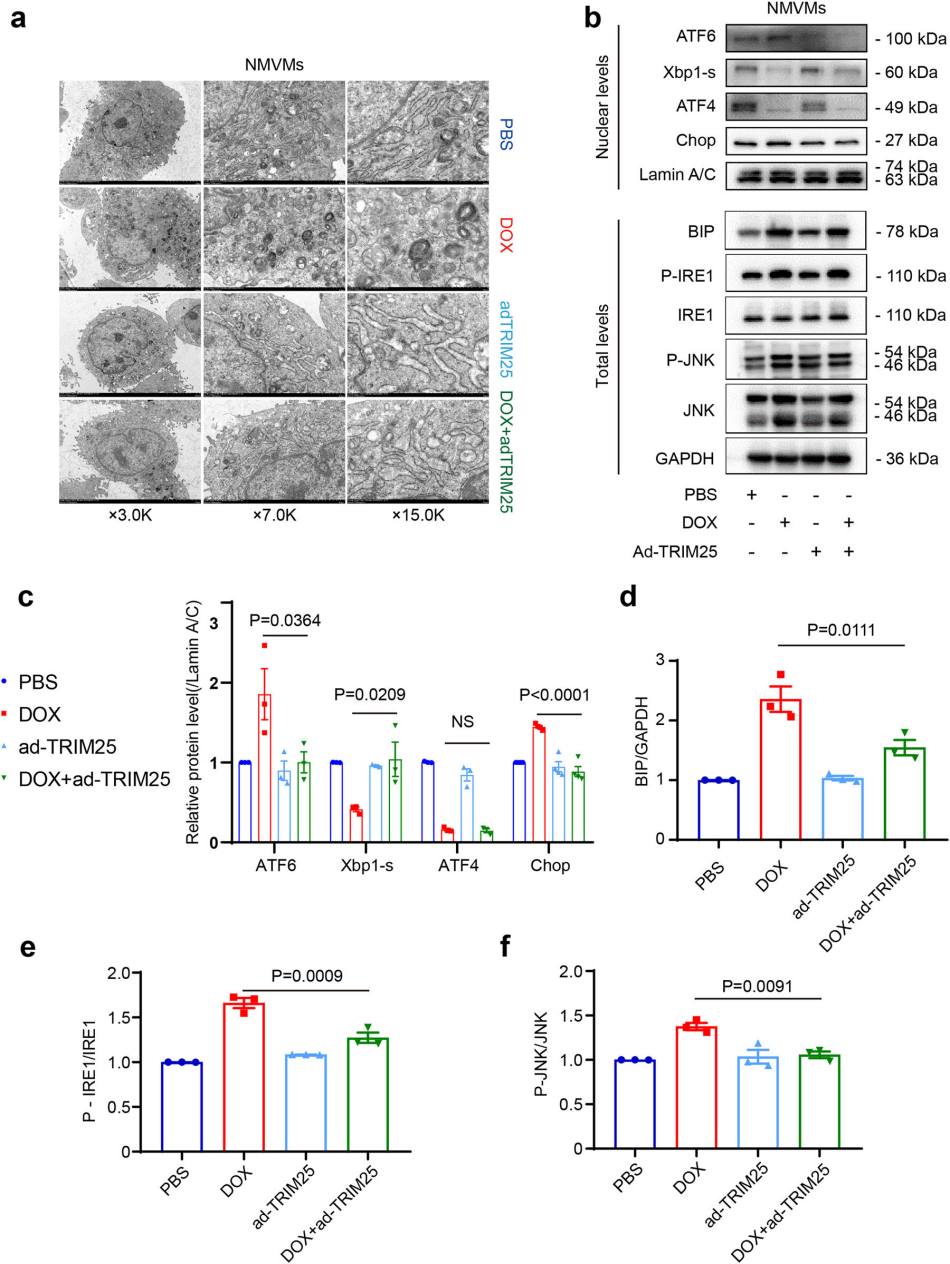
TRIM25 overpression by adenovirus attenuates DOX-induced apoptosis and endoplasmic reticulum stress in cardiomyocytes. **a** DOX-induced NMVMs were transfected with Ad-TRIM25 and assessed by transmission electron microscopy. **b** Representative western blotting (above) and the corresponding quantified graph (**c**–**f**) (below) of Atf6, Atf4, XBP1-s, and Chop in the nuclear fraction and BIP, P-IRE1, IRE1, P-JNK, and JNK in the total protein of NMVMs. Data represent the means ± SEM (*n* = 3–5). *P* values were analyzed by one-way ANOVA test with Bonferroni *post hoc* test (**c**, **d**, **e**, **f**). Ad-TRIM25, adenovirus-TRIM25; NS not significant, NMVM neonatal mouse ventricular myocytes.

We performed transmission election microscopy to observe the ER lumen in the primary cardiomyocytes treated with DOX. The distention of the ER was augmented by DOX treatment (Fig. 5a), and we found that even a single injection of DOX could result in constant distention of the ER in vivo (Supplementary Fig. 12). BIP/GRP78, as sensor and effector for ER stress, is considered as ER stress marker. Western blotting revealed that BIP was elevated in DOX-treated cardiomyocytes and reversed by TRIM25 overexpression. The UPR signaling pathways that govern ER proteostasis, was upregulated by DOX treatment. Additionally, the phosphorylation of JNK pathway was increased after DOX treatment, suggested the maladaptive UPR was also involved in DOX-induced apoptosis (Fig. 5b). Conversely, TRIM25 overexpression attenuated the distention of the ER, as well as the expression of ATF6 and CHOP in DOX-treated cardiomyocytes. The spliced form of XBP-1 (XBP-1s), a crucial transcription factor in mitigating ER stress, was downregulated after DOX treatment. In summary, TRIM25 mitigated DOX-induced ER stress in cardiomyocytes (Fig. 5b, c, d, e). Surprisingly, ATF4, which was elevated in failing hearts in humans, was significantly downregulated in DOX-treated cardiomyocytes and not affected by TRIM25 overexpression (Fig. 5f). ATF4 has pleiotropic functions to reduce the levels of unfolded and misfolded proteins in the ER; however, it can also control CHOP expression to induce cell death. The function of ATF4 in DOX-induced cardiotoxicity needs to be elucidated further in future studies.

### TRIM25 suppresses the UPR by facilitating p85α degradation

**Fig. 6:**
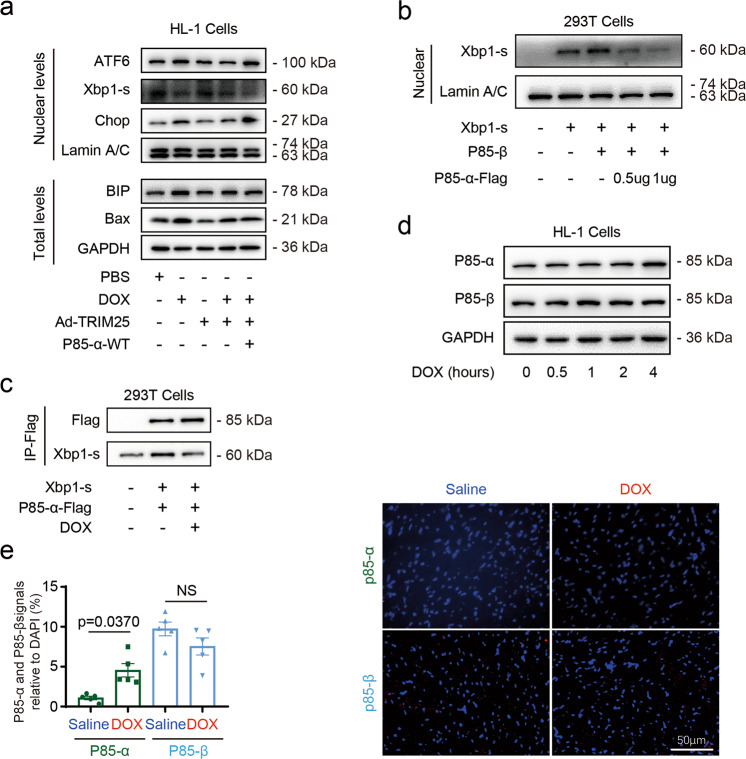
TRIM25 increases nuclear transport of XBP-1s by ubiquitinating p85α. **a** Nuclear protein amounts of ATF6, XBP-1s, Chop, and Lamin A/C and total protein of BIP, Bax, and Gapdh in cardiomyocytes infected with Ad-TRIM25 and p85α (plasmid, 1 μg/ml) for 48 h and then treated with DOX (1 uM) for 12 h. Western blot analysis of the levels of Bax in HL-1 cells. **b** Nuclear protein amounts of XBP-1s and Lamin A/C in 293 T cells infected with XBP-1s (2 μg), p85β (2 μg), and with increasing doses of p85α (0.5 μg, 1 μg). **c** Flag and XBP-1s blotting in Flag immunoprecipitates of 293 T cells treated with XBP-1s, p85α-Flag, and DOX (1 uM). **d** Representative western blot images for p85α and p85β in HL-1 cells stimulated with a constant dose of DOX (1 uM) for 0, 0.5, 1, 2, and 4 h. **e** Representative images and quantification of p85α and p85-β expression in hearts sections from mice with one injection of DOX (5 mg/kg); p85α and p85β (red), DAPI for nuclei (blue). Scale bar, 50 μm in e. *N* = 5 animals/group; Data represent the means ± SEM. (*n* = 5). NS not significant.

As TRIM25 is a well-known E3 ligase [26], we further investigated whether TRIM25 regulates UPR via the degradation of p85α specifically. Expectedly, TRIM25 significantly attenuated DOX-induced apoptosis and the protection function of TRIM25 was slightly altered upon p85α knockdown in the cardiomyocytes (Supplementary Fig. 13). Additionally, TRIM25 significantly decreased ATF6, CHOP, and BIP accumulation in the cardiomyocyte nucleus, whereas XBP-1s was upregulated by TRIM25 (Fig. 6a). Additionally, western blotting revealed that p85α overexpression markedly reversed the beneficial effect of TRIM25 overexpression on diminishing ER stress, further confirming the therapeutic effect of TRIM25 attributed to the degradation of p85α.

The regulatory subunits of PI3K, p85α, and p85β are master regulators of XBP-1s and UPR [27]. As reported, monomers of p85α and p85β could interact with as well as increase the nuclear translocation of XBP1-s under normal circumstances. To examine the association between p85α and p85β together with the nuclear translocation of XBP-1s, we increased p85α input while keeping p85β and XBP-1s expression constant. Western blotting revealed that the nuclear translocation of XBP-1s was reduced when the p85α expression was increased (Fig. 6b). The immunoprecipitation of the Flag tag decreased XBP-1s expression, confirming an interaction between p85α and XBP-1s. Furthermore, this interaction was weakened by DOX treatment, which also contributed to further decrease of XBP-1s’ nuclear translocation and finally led to ER stress augmentation (Fig. 6c). Immunofluorescence analysis revealed that the expression of p85β was markedly more than that of p85α in cardiomyocytes. Consistent with the immunofluorescence analysis, western blotting also revealed that DOX treatment resulted in an increase of p85α while it had no obvious effect on the expression level of p85β. This indicates that after DOX treatment, there was a decrease in the expression of nuclear translocation of XBP-1s (Fig. 6d, e).

### P85α overexpression or enhanced ER stress in doxorubicin-treated mice abolishes TRIM25-induced cardioprotection

Given that the relationship between ER stress and DOX-induced cardiotoxicity has not been widely explored [28, 29], we examined the role of an anti-ER stress chemical chaperone, tauroursodeoxycholic acid (TUDCA) in DOX-induced cardiomyopathy (Supplementary Fig. 14a). We found that TUDCA could improve heart function and myocardial atrophy significantly (Supplementary Table 5). Moreover, TUDCA strongly decreased Anp and Bnp levels (Supplementary Fig. 14b, c, d, e). Similarly, hematoxylin-eosin staining revealed that the heart’s cross-sectional area was improved by TUDCA treatment and Masson staining showed that TUDCA treatment could also decrease the cardiac fibrosis area (Supplementary Fig. 15a, b).

**Fig. 7:**
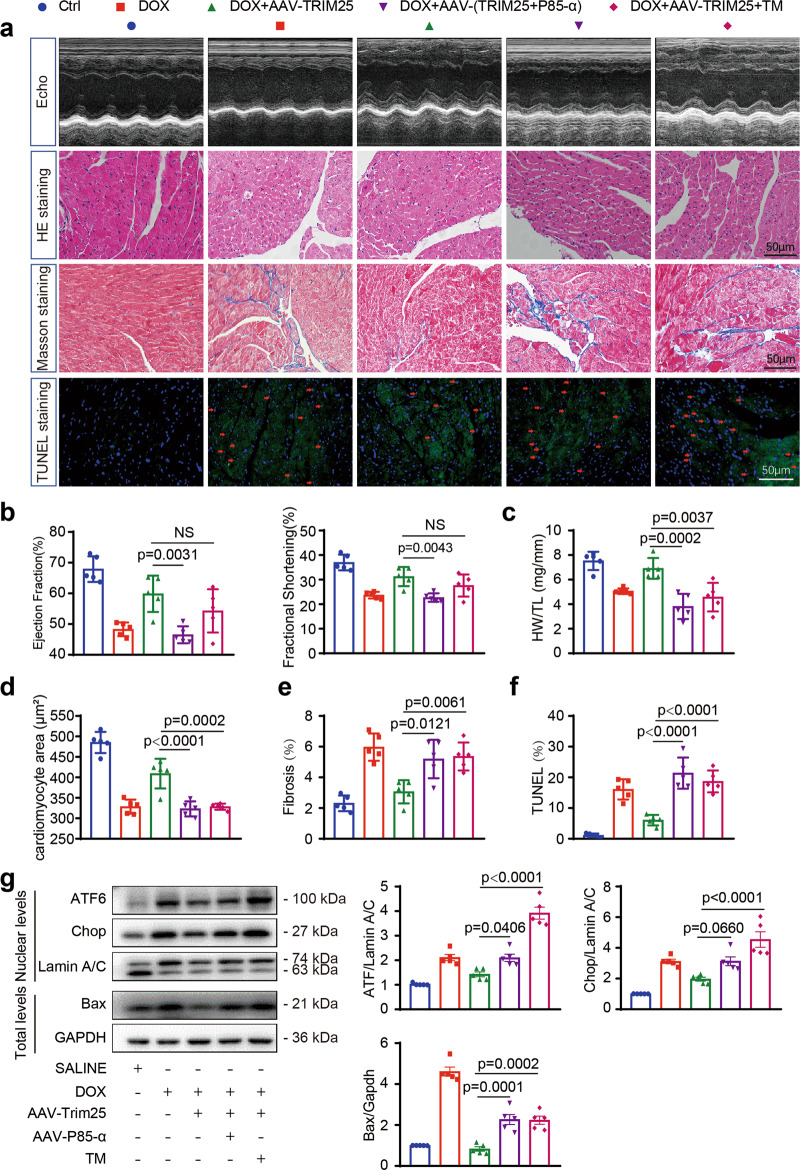
Pharmacological augmentation of endoplasmic reticulum stress or p85α overexpression abolishes cardioprotection in TRIM25 OE mice. TRIM25 or p85α overpression (OE) mice were injected by adeno-associated virus 9 via the tail vein, and after 4 weeks, they were treated with a cumulative dose of 20 mg/kg DOX by 4 weekly injections (5 mg/kg on days 0, 7, 14, and 21) and TM (tunicamycin) (2 mg/kg on days 0, 7, 14, and 21). **a** Representative M-mode echocardiographic images as well as H&E, Masson, and TUNEL staining in heart sections from CON, DOX, AAV-TRIM25, AAV-p85α, and TM-administered mice. **b** M-mode images from three cardiac cycles for each animal were used to calculate ventricular measurements: ejection fraction, fractional shortening. **c** The HW/TL (heart weight/tibia length) ratio as a cardiac atrophy index of mice. (**d**, **e**, **f**) Relative quantification of cardiomyocyte area, fibrosis, and TUNEL-positive nuclei per field in heart sections from CON, DOX, AAV-TRIM25, AAV-p85α, and TM-administered mice. **g** Nuclear protein amounts of ATF6, CHOP, and Lamin A/C in mice hearts treated with DOX, AAV-TRIM25, AAV-p85α, and TM. Western blot and relative quantification for Bax in heart tissue from mice infected with DOX, AAV-TRIM25, AAV-p85α, and TM. *N* = 5 animals/group; Values represent mean ± SEM. Scale bar, 50 μm in a. TM tunicamycin.

**Fig. 8:**
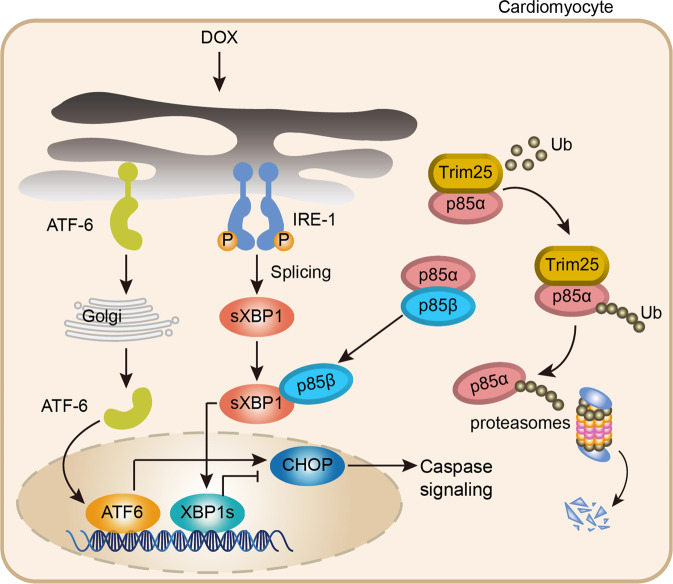
Schematic diagram of the molecular mechanisms underlying TRIM25-regulated DOX-induced cardiotoxicity. A schematic model illustrating that TRIM25 regulates the p85α/XBP-1s/CHOP signaling pathway under ER stress to decrease myocardial apoptosis. TRIM25, which is downregulated after treatment with DOX, could alleviate ER stress by targeting p85α for ubiquitination and degradation, leading to increased XBP-1s transferring to the nucleus.

The protective effects of TRIM25 overexpression with respect to DOX-treated cardiotoxicity are likely linked to p85α downregulation and its role in the UPR. To test this hypothesis, we employed an AAV9 system to overexpress p85α in vivo via tail vein injection. Western blotting and quantitative polymerase chain reaction analyses confirmed p85α overexpression in the heart of AAV-p85α-injected mice (Supplementary Fig. 16). As expected, infection with TRIM25 and p85α significantly blunted the cardioprotective effects of TRIM25 against DOX-induced deterioration of cardiac function (Fig. 7a, b). Similar results were observed when ER stress was enhanced by a pharmacological agent, tunicamycin (TM). AAV-p85α-injection or TM could impair cardiac contractility (Supplementary Table 6). In the mouse model, cardiac atrophy enhanced by AAV-p85α-injection or TM was evident in heart weight/tibia length ratios and cardiomyocyte size (Fig. 7c, d). Masson and TUNEL staining illustrated that DOX-induced cardiac fibrosis and apoptosis was increased by AAV-p85α-injection or TM (Fig. 7e, f). Western blotting revealed the role of p85α in UPR and apoptosis under conditions of irreversible ER stress. Taken together, these results indicate the protective effects of downregulation of p85α against DOX-induced cardiotoxicity caused by TRIM25 and its enhancement in ER stress and apoptosis (Fig. 7g). Fig. 8.

## Discussion

This study defined TRIM25 as a crucial factor of DOX-induced cardiotoxicity and demonstrated the previously unrecognized biological function of TRIM25 in inhibiting ER stress. During the development of pathological cardiotoxicity, the expression level of p85α was upregulated by a ubiquitin-proteasome system involving TRIM25. Mechanistically, our results demonstrated that TRIM25-mediated ubiquitination of p85α at K506 and K511 played a crucial role in determining the expression level of p85α, which further regulated the ER stress induced by DOX. We also confirmed the role of ER stress in DOX-induced cardiotoxicity using both in vivo and in vitro models. By attenuating ER stress, cardiotoxicity could be effectively prevented. Herein, we demonstrated that TRIM25 mitigated cardiotoxicity through the ubiquitination of p85α, resulting in the regulation of UPR signaling and apoptosis.

Although various molecular mechanisms related to DOX-induced cardiotoxicity have been reported previously, most of them were attributable to the overproduction of ROS in cardiomyocytes [25, 30]. Dexrazoxane as an anti-ROS therapy that has been used modestly in clinical settings and proved to increase risk of developing secondary malignancies, suggesting that the understanding of cardiotoxicity remains incomplete [31]. Our results also revealed a new mechanism that the disorder level of ubiquitin E3 ligases could contribute to DOX-induced cardiotoxicity. Members of the TRIM protein family participate in multiple biological processes through their ubiquitination activity, such as cell proliferation, differentiation, development, and innate immunity [16]. Several studies have reported that the overexpression of MG53, a muscle-specific TRIM family protein, can attenuate hypoxia-induced cardiomyocyte death and treat some aspects of the disease in mouse models of muscular dystrophy [32]. Regarding metabolism, Trim16 could ameliorate nonalcoholic steatohepatitis by promoting the degradation of phospho-TAK1 [17]. TRIM25 has been reported to promote cell survival by ubiquitinating and degrading Keap1 in hepatocellular carcinoma [33]. Wang et al. have reported that a significant decrease in TRIM25 expression at the early stage of doxorubicin treatment and TRIM25 could play a role in the reversal of doxorubicin-induced cardiotoxicity in both in vivo and in vitro models, which is consistent with our results [34]. In contrast, we treated cardiomyocytes with doxorubicin at a concentration of 1 µM, which is closer to the concentration of doxorubicin exposure of cardiomyocytes in clinical chemotherapy, therefore, our results indicated important alterations and effects of endoplasmic reticulum stress in doxorubicin-induced cardiotoxicity. Furthermore, we have found that, as a ubiquitin E3 ligase, TRIM25 participated in degrading p85α and in turn modified UPR signaling, resulting in the amelioration of cardiomyocyte ER stress and apoptosis.

The role of ER stress in cardiac diseases is vital with respect to multiple pathological developments [19, 35]. Notably, the unique pathological change of DOX-induced ER distention was observed in our study as well as in a previous report by Minotti [36], whereby the distension could last for more than 8 weeks even with a single injection of DOX in vivo. Whether this pathological ER change is responsible for the increased long-term cardiac mortality in patients with cancer remains unclear. Furthermore, our findings revealed that DOX augmented ER stress in cardiomyocytes and that this increase is harmful to the heart in vivo. Attenuating ER stress via the injection of TUDCA, an anti-ER stress chemical chaperone, is a useful method to prevent DOX-induced cardiomyopathy.

Regarding the accumulation of ER stress, the UPR represents a key adaptive response to regulate improperly folded proteins and restore ER homeostasis [37, 38]. As reported previously, signaling including the protein kinase RNA (PKR)-like ER kinase (PERK)-eukaryotic translation initiation factor 2 A (eIF2a)‑activating transcription factor (ATF) 4‑C/EBP homologous protein (CHOP) pathway, transcription factor 6 (ATF6)-CHOP pathway, and inositol-requiring kinase 1 (IRE1)- X‑box binding protein 1 (XBP1) pathway control the UPR [39]. Sustained UPR activation can lead to a maladaptive condition and eventually, cell death. In the current study, the nuclear levels of ATF6, XBP-1s, and CHOP revealed the role of UPR in DOX-induced cardiotoxicity. The impaired XBP-1s nuclear translocation induced by DOX may be one of the reasons for maladaptive UPR. Yucel et al. reported that XBP-1s was an important transcription factor in regulating UPR target genes involved in protein folding [40]. XBP-1s nuclear translocation could increase ER-resident protein mesencephalic astrocyte-derived neurotrophic factor transcription and reduce ER stress [41]. Inactivation of mesencephalic astrocyte-derived neurotrophic factor induces cell death by upregulation of the pro-apoptotic mediator, CHOP [42]. A large amount of evidence has indicated the beneficial effect of XBP-1s in cardiovascular diseases. Wang et al. also indicated that XBP-1s expression was reduced in both human and rodent cardiac tissues under heart failure, and that XBP-1s overexpression prevented the development of cardiac dysfunction [43]. Furthermore, XBP-1s couples the UPR to the HBP (hexosamine biosynthetic pathway) to protect cells under stress, which protect the heart from ischemia/reperfusion damage [44, 45]. Interestingly, our study and a previous study by Park et al. showed that monomers of p85α or p85β could increase the nuclear translocation of the spliced XBP-1 separately [27]. However, another study suggested that there was a competitive interaction among p85α, p85β, and XBP-1s under normal circumstances, whereby p85α and p85β form heterodimers. However, the homeostasis among p85α, p85β, and XBP-1s was broken by the expression of E3 enzyme which regulated the expression level of p85α, P85β, and XBP-1s induced by DOX. Our data also demonstrated that DOX could further decrease the transport of XBP-1s by decreasing the interaction between p85α and XBP-1s. Previous research has shown that there is a reduced accumulation of UPR proteins such as BIP (the hallmark of ER stress), CHOP, and ATF6 in the absence of p85α in fibroblasts treated with tunicamycin [46]. In our study, the therapeutic effect of TRIM25 overexpression, would be attributable, at least in part, to p85α downregulation.

In summary, our study provides evidence that TRIM25 mediated DOX induced ER stress via its effects on the p85α-XBP-1s pathway. Our findings, therefore, highlight a novel role of TRIM25 in DOX-induced cardiotoxicity. Targeting the p85α-XBP-1s pathway by regulating the ubiquitin E3 ligase, TRIM25, may provide a potential strategy for patients undergoing chemotherapy-based regimens.

## Supplementary information

Author Contribution Statement

Material and Methods

Original Data File

checklist

## Data Availability

The data in the study are available upon reasonable request.
