# Supplementary material for: Tripartite motif 25 ameliorates doxorubicin-induced cardiotoxicity by degrading p85α
Source: Cell Death Dis. 2022 Jul 23;13(7):643. doi: 10.1038/s41419-022-05100-4 (PMC9308790; doi:10.1038/s41419-022-05100-4)
Supplement: Supplementary file 1 — Author Contribution Statement [file 41419_2022_5100_MOESM1_ESM.docx]

L. Cheng and Y. Shen conceived the study. J. Zhao and Y. Shen designed the experiments. Y. Shen, H. Zhang, Y. Ni, X. Wang, Y. Chen, J. Chen, Y. Wang, J. Lin, and Y. Xu performed the experiments and analyzed the data. Y. Shen wrote the initial draft of article. L. Cheng and J. Zhao revised the article.
