# Supplementary material for: Tripartite motif 25 ameliorates doxorubicin-induced cardiotoxicity by degrading p85α
Source: Cell Death Dis. 2022 Jul 23;13(7):643. doi: 10.1038/s41419-022-05100-4 (PMC9308790; doi:10.1038/s41419-022-05100-4)
Supplement: Supplementary file 2 — Material and Methods [file 41419_2022_5100_MOESM2_ESM.docx]

**Supplementary Material**

**Tripartite motif 25 ameliorates Doxorubicin-induced cardiotoxicity by degrading p85α**

Yihui Shen, Hui Zhang, Yangyue Ni, Xuejun Wang, Yifan Chen, Jiahui Chen, Yan Wang, Jinyi Lin, Yuchen Xu, Jian-Yuan Zhao, Leilei Cheng

**Supplementary Fig. 1-16**

**Supplementary table 1-6**


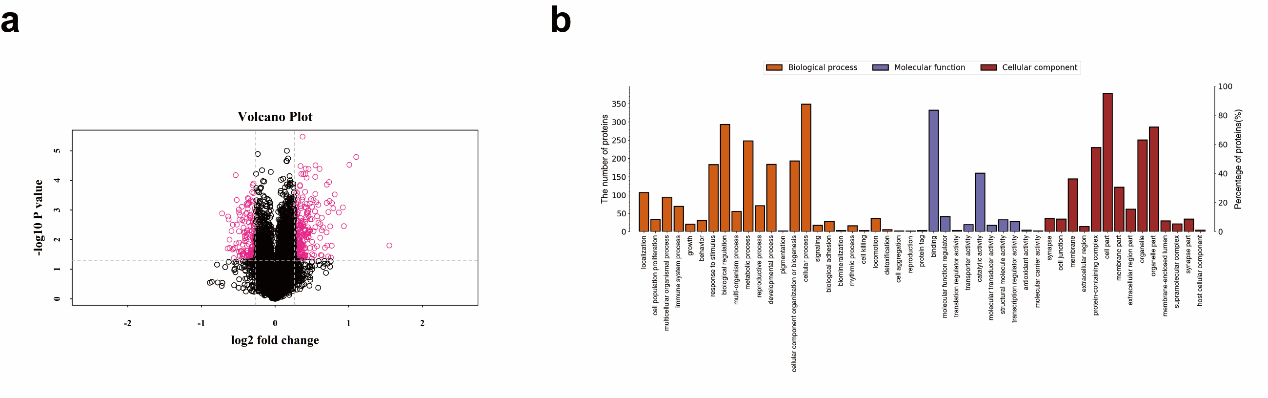


**Supplementary Fig. 1. Proteomics and bioinformatics analysis of the proteins induced by DOX in cardiomyocytes.**

(**a**) The volcano plot for differentially expressed proteins. The horizontal axis represents the fold change, and the vertical axis represents the adjusted p-value.

(**b**) Gene Ontology (GO) revealed that DOX treatment activated the differentially proteins in terms of biological process, cellular component and molecular function.


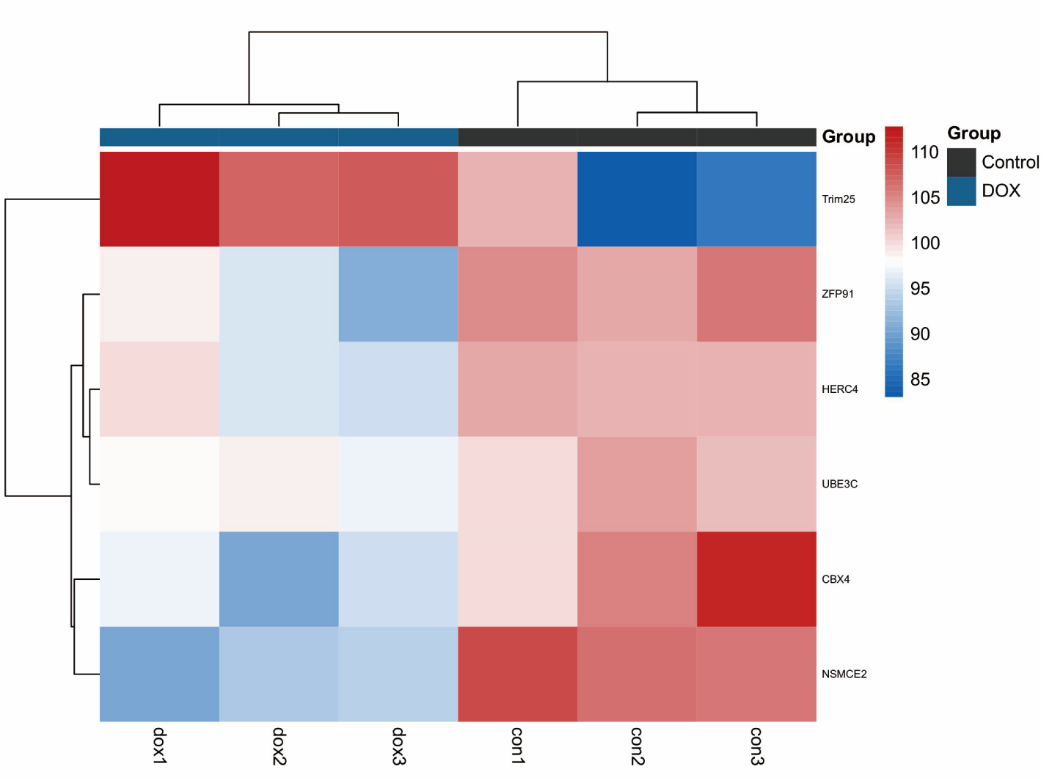


**Supplementary Fig. 2. Relative fold change of differentially expressed E3 ligases, which were consistent in both cell and tissue models.**

Hierarchical clustering heatmap of differentially expressed E3 ligases, which met the criteria of the screen; namely, TRIM25, CBX4, HERC4, NSMCE2, UBE3C, and ZFP91 in proteomics results.


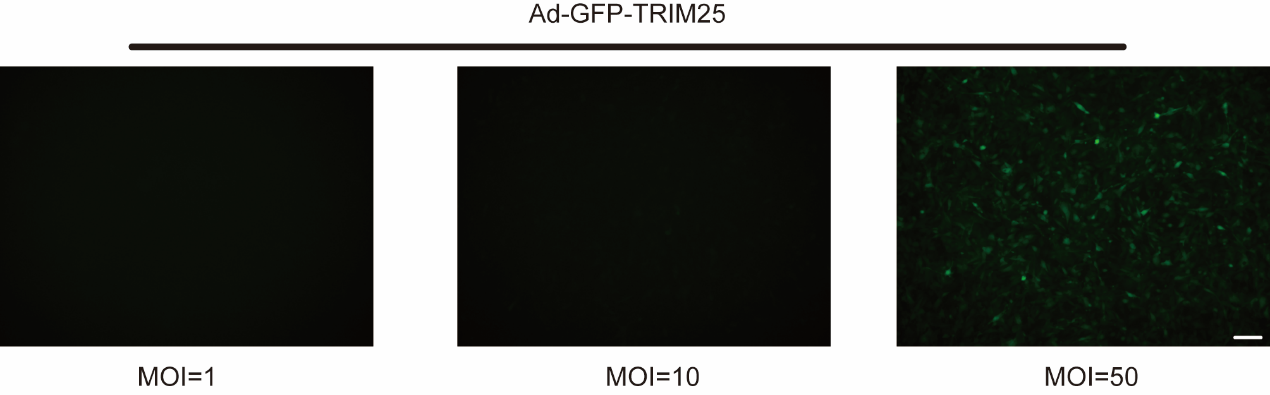


**Supplementary Fig. 3. Adenovirus expression efficiency on TRIM25 in NRCMs**

Adenovirus encoding green fluorescent protein was detected 48h after the adenovirus transfection in NRCMs. Scale bar = 100 μm.


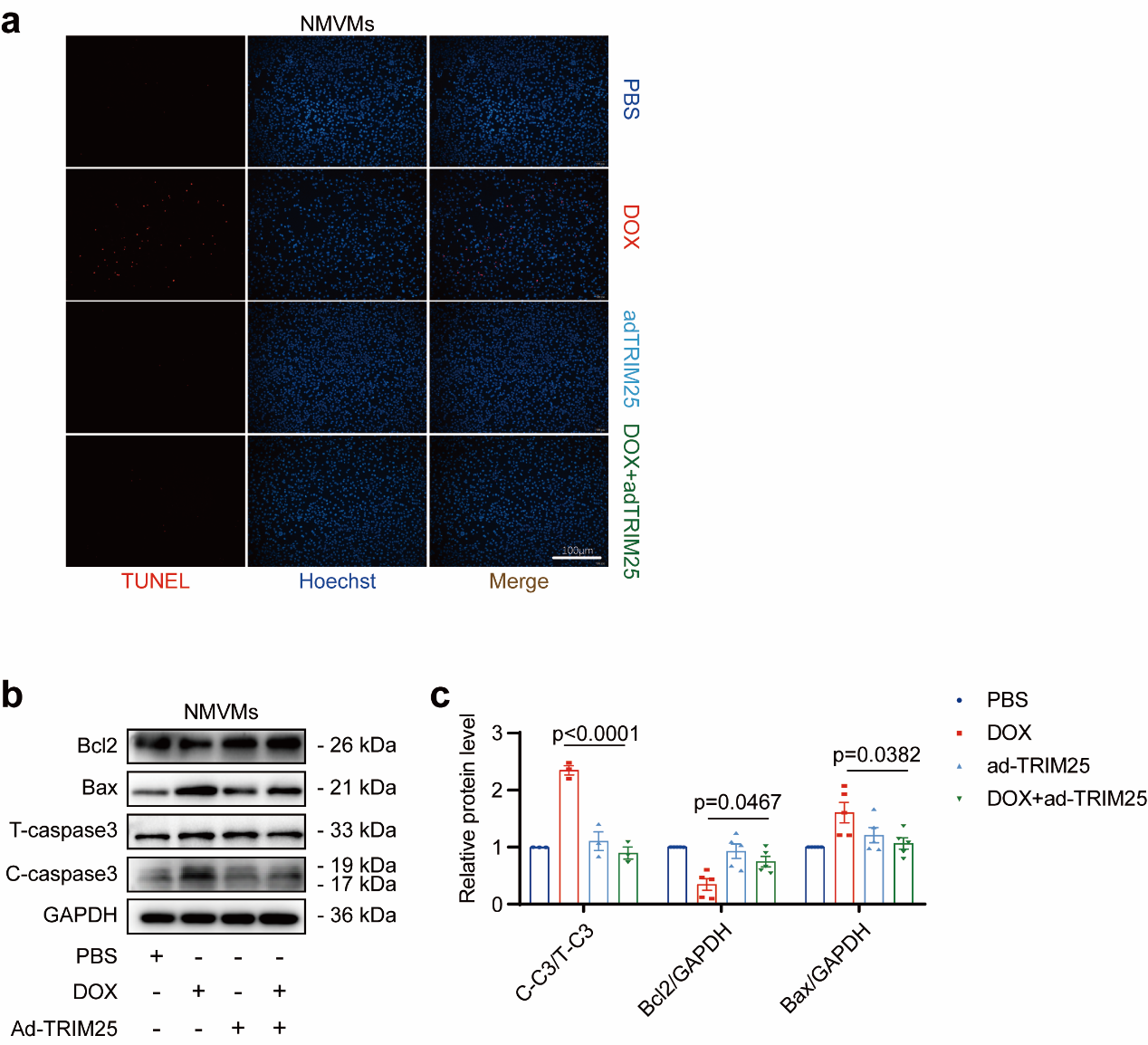


**Supplementary Fig. 4. TRIM25 overexpression by adenovirus attenuates DOX-induced apoptosis in cardiomyocytes**

(**a**) TUNEL staining (red) indicates cardiomyocyte apoptosis, and Hoechst staining (blue) indicates cardiomyocyte nuclei. Merged TUNEL and Hoechst staining images demonstrate apoptotic cardiomyocyte nuclei (**b**-**c**) Representative western blot (left) and the relative quantification (right) of Bcl2, Bax, Total caspase-3, Cleaved caspase-3 in cardiomyocytes are shown. Data represent the means ± SEM (n=3-5). P values were analyzed by one-way ANOVA test with Bonferroni post hoc test. Scale bar, 100μm in A. Ad-TRIM25 indicates adenovirus-TRIM25; NS, not significant; C-C3, Cleaved caspase-3; and NMVM, neonatal mouse ventricular myocytes.


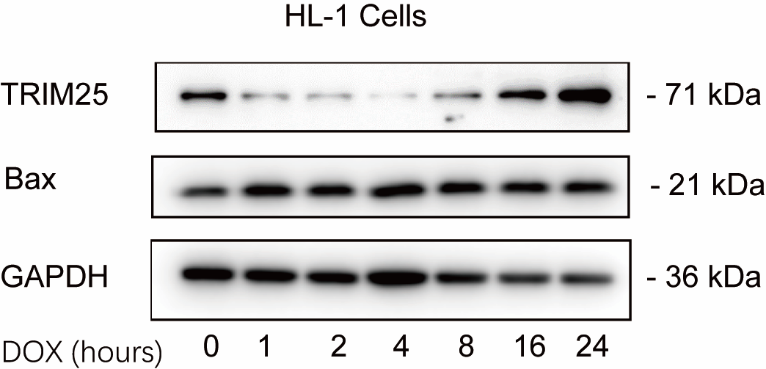


**Supplementary Fig. 5. Representative western blot of TRIM25 in HL-1 cells treated by DOX with time-dependent manner**

Representative western blot of Bax, TRIM25, and GAPDH in DOX-treated cardiomyocytes with time-dependent manner are shown.


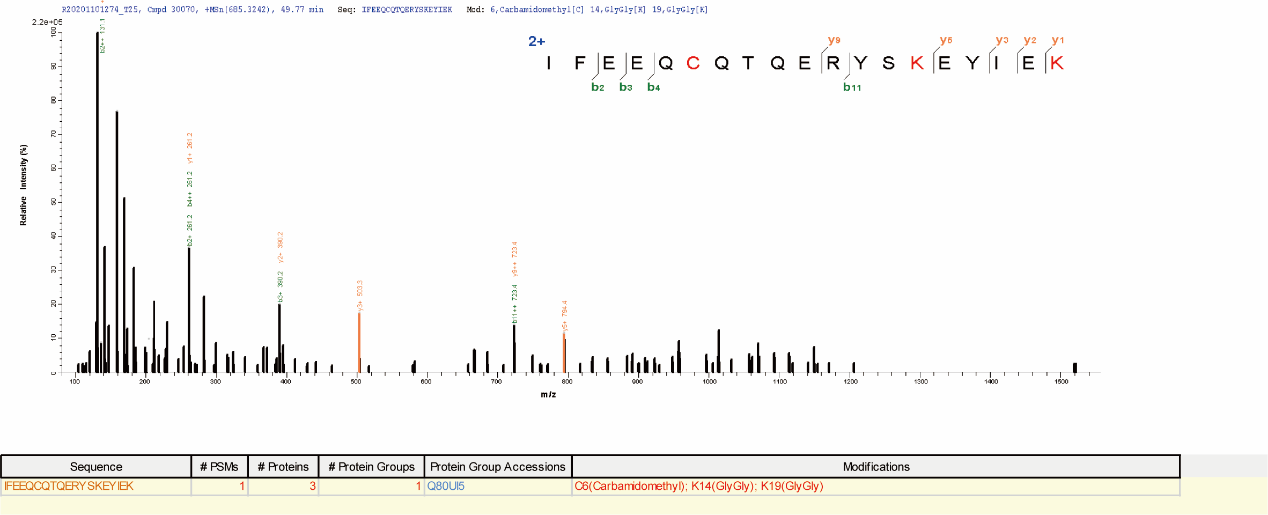


**Supplementary Fig. 6. Lysine site of ubiquitination of p85α in cardiomyocyte after overexpression of TRIM25**

LC-MS/MS scans of Ubiquitination site of p85α in cardiomyocyte were performed. The representiative glygly-group was detected in the peptide fragmentation of p85α. Sequence: IFEEQCQTQERYSKEYIEK.


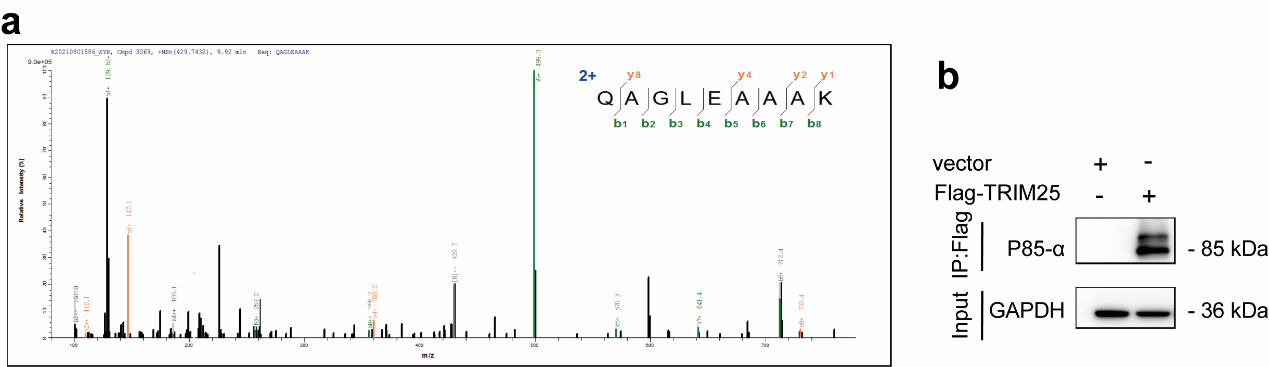


**Supplementary Fig. 7. Interaction between p85α and TRIM25 in HEK 293T cells**

(**a**) Mass spectrometry was performed to screen the interacted protein with p85α. Flag-p85α was overexpressed in HEK 293T cells. The interaction proteins with p85α were immuno-precipitated by Flag antibody. TRIM25 was screened out. (**b**) Representative coimmunoprecipitation analysis of p85α and TRIM25 in HEK 293T cells. The interaction between p85α and TRIM25 was confirmed by coimmunoprecipitation analysis of p85α and TRIM25.


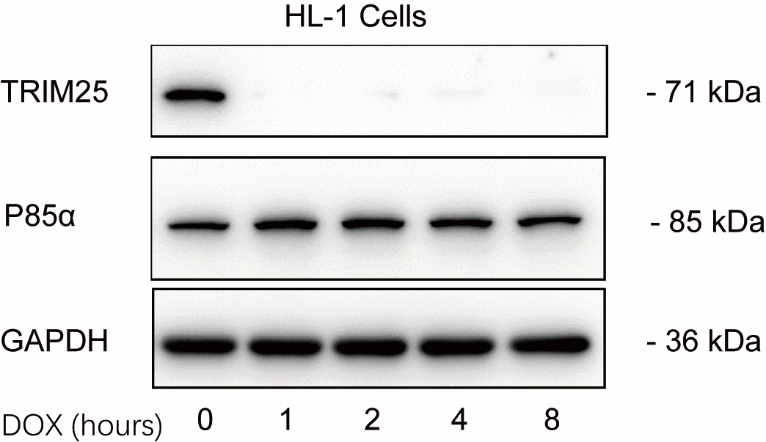


**Supplementary Fig. 8. Representative western blot of TRIM25 in HL-1 cells treated by DOX with time-dependent manner**

Representative western blot of p85α, TRIM25, and GAPDH in DOX-treated cardiomyocytes with time-dependent manner are shown.


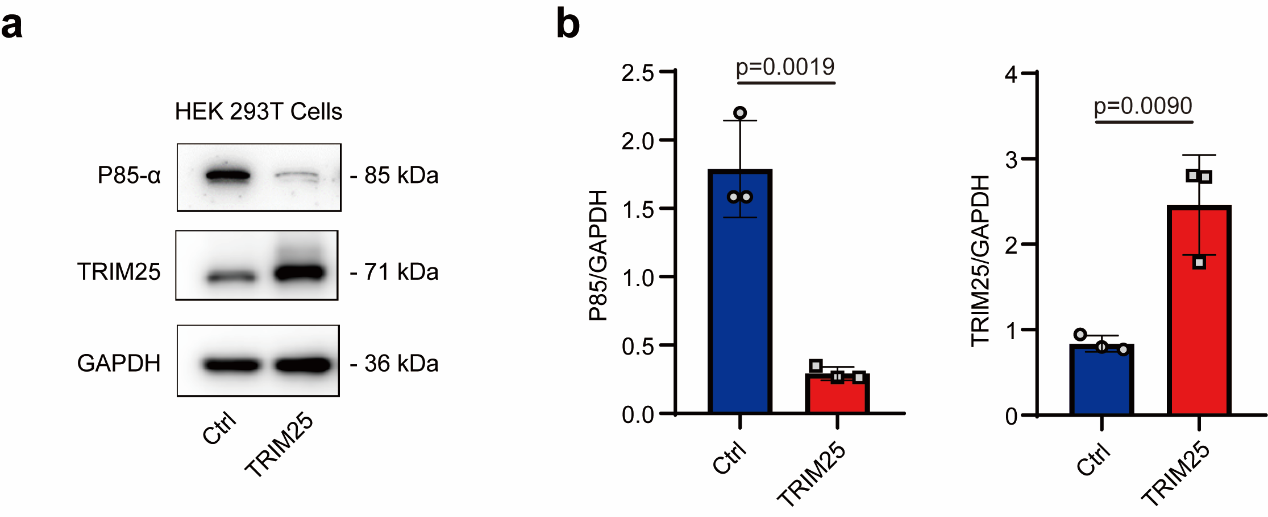


**Supplementary Fig. 9. Western blot analysis of p85α and TRIM25 levels**

Western blot analysis of p85α and TRIM25 levels in HEK 293T cells transfected with TRIM25 overexpression plasmids (TRIM25-WT) or control plasmid. Data represent the means ± SEM (n = 3). P values were determined by unpaired 2-tailed Student t test.

**
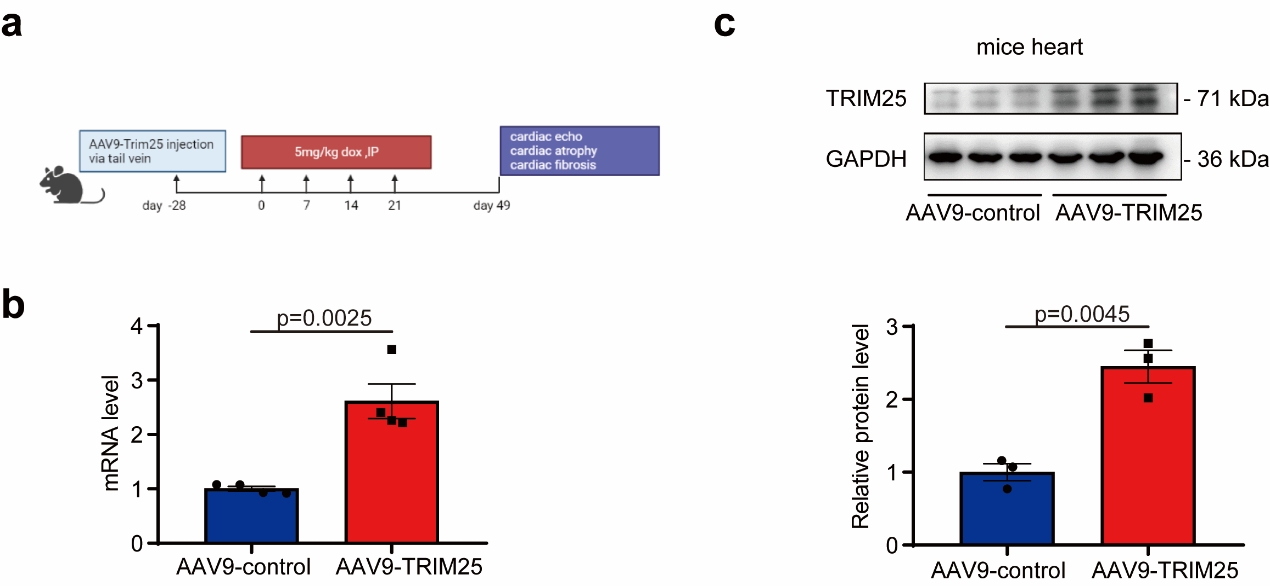
 Supplementary Fig. 10. Adeno-associated virus 9 expression efficiency on TRIM25 in mouse heart**

(**a**) Schematic illustration showing chronic models of DOX-induced cardiotoxicity,

(**b**) Representative western blot image showing TRIM25 protein level in the heart of DOX-treated mice injected with AAV-Control or AAV-TRIM25, which were performed in mice to test the effect of overexpression of TRIM25 in heart.

(**c**) Representative mRNA level of TRIM25 in heart of the mice.

Data represent the means ± SEM (n = 3-4), P values were determined by unpaired 2-tailed Student t test.


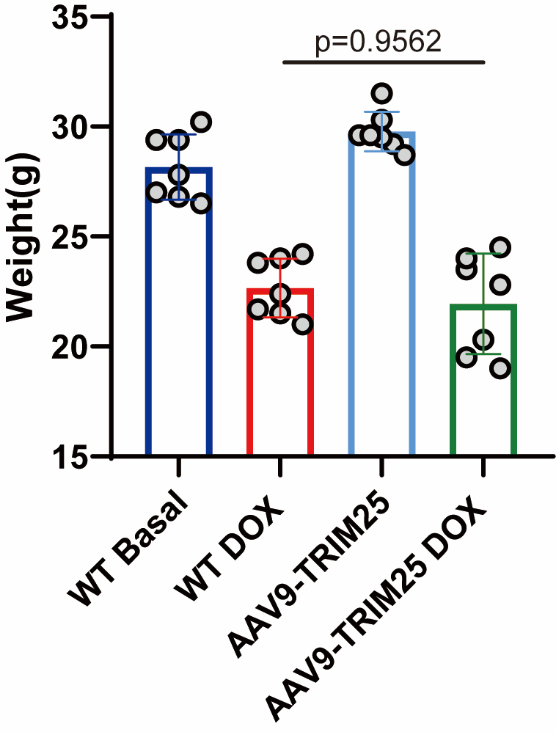


**Supplementary Fig. 11. The weight of mice treated by DOX and TRIM25 overpression by adeno-associated virus**

TRIM25 overpression by adeno-associated virus did not affect DOX-induced loss of weight in mice. Values represent mean ± SEM. n=7 independent experiments. P values were analyzed by one-way ANOVA test with Bonferroni post hoc test.


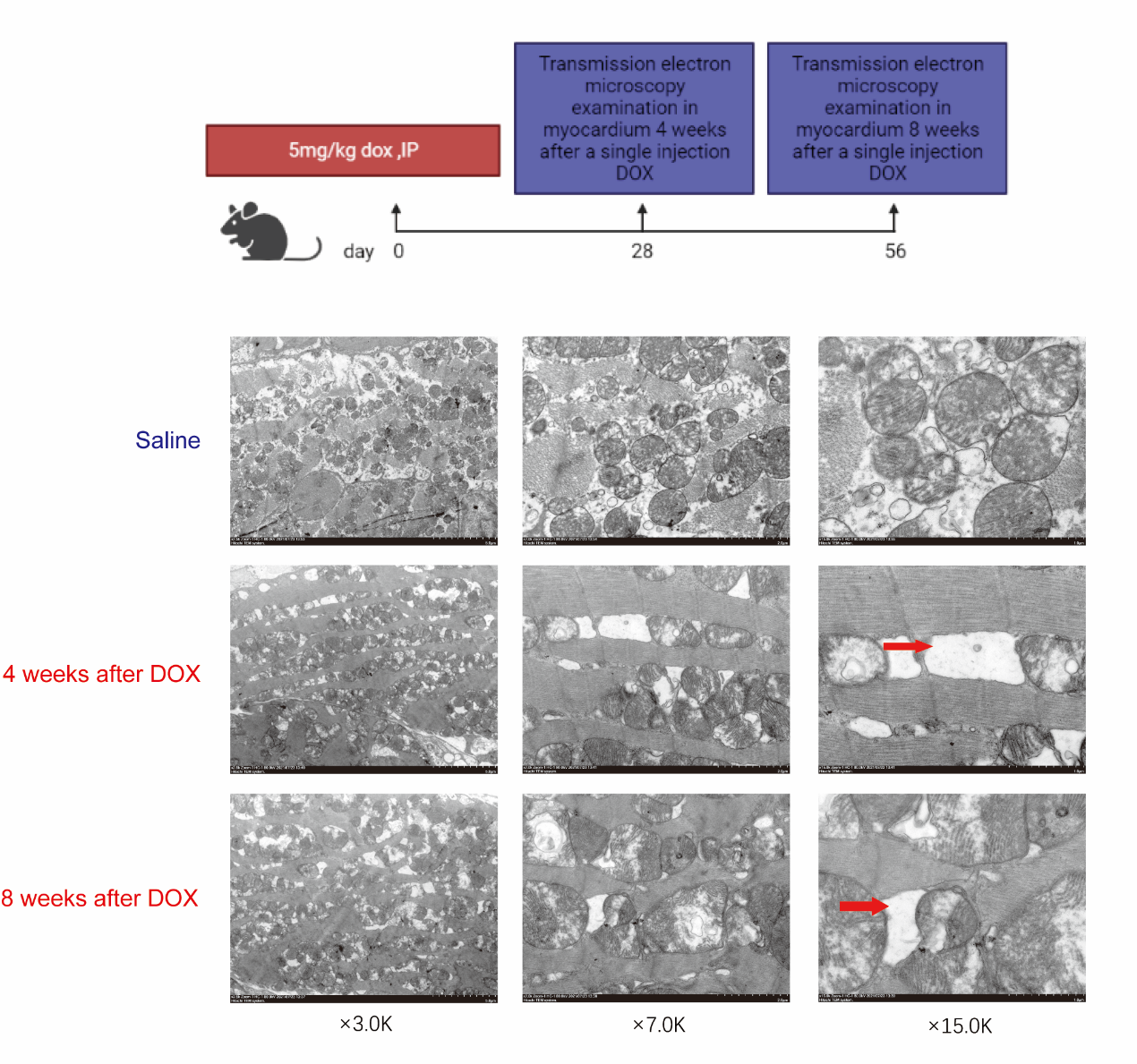


**Supplementary Fig. 12. Transmission electron microscopy detected the ultrastructure of endoplasmic reticulum in mouse heart**

Transmission electron microscopy showed the ultrastructure of ER in the left ventricular. The ultrastructure of ER (endoplasmic reticulum) in heart of mice treated with DOX was detected distention for more than 8 weeks. The ER structure is pointed by arrows in the right images. The arrows indicate endoplasmic reticulum.


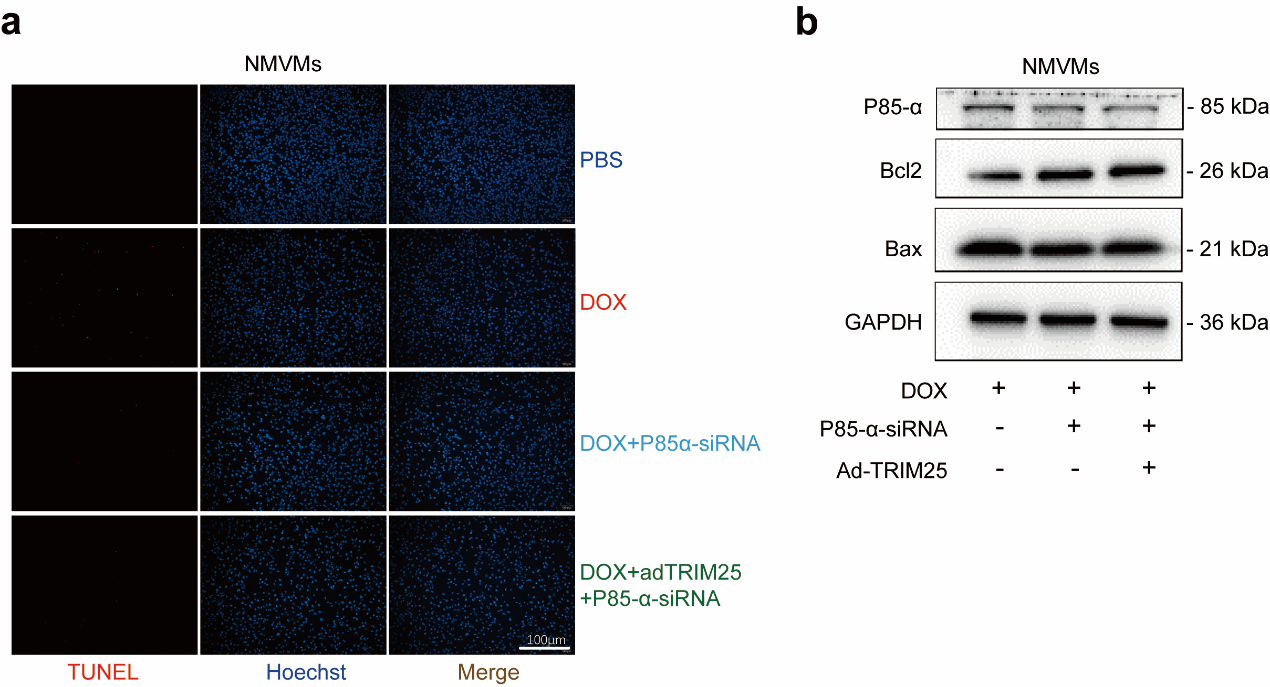


**Supplementary Fig. 13. TRIM25 overexpression by adenovirus slightly alters DOX-induced apoptosis upon p85α knockdown in cardiomyocytes**

(**a**) TUNEL staining (red) indicates cardiomyocyte apoptosis, and Hoechst staining (blue) indicates cardiomyocyte nuclei. Merged TUNEL and Hoechst staining images demonstrate apoptotic cardiomyocyte nuclei (**b**) Representative western blot of p85α, Bax, Bcl2 in cardiomyocytes are shown. Scale bar, 100μm in a. Ad-TRIM25 indicates adenovirus-TRIM25; NMVM, neonatal mouse ventricular myocytes.


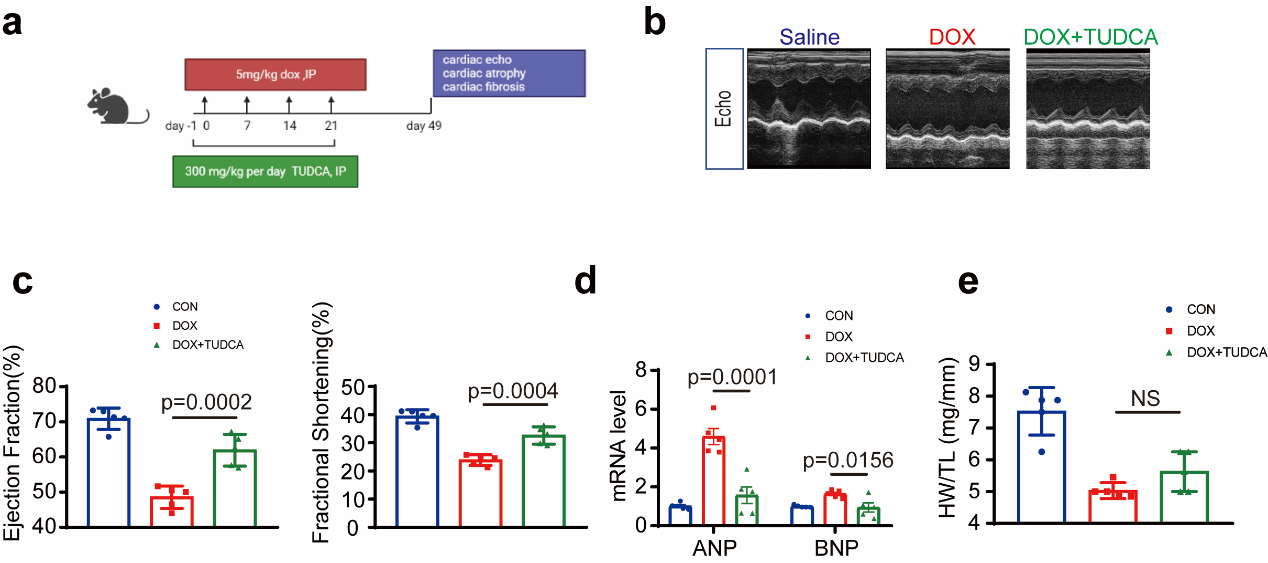


**Supplementary Fig. 14. Suppression ER stress attenuates the DOX-induced cardiac dysfunction by chemical chaperone, TUDCA *in vivo***

(**a**) Schematic illustration showing chronic models of DOX cardiotoxicity, which were performed in mice to test the effect of injection of TUDCA *in vivo*.

(**b**) Representative M-mode echocardiographic images of WT and TUDCA hearts 4 weeks after the first DOX injection.

(**c**) M-mode images from for each animal were used to calculate ventricular measurements: ejection fraction, fractional shortening.

(**d**) Relative quantification of cardiac Anp and Bnp mRNA expression levels.

(**e**) The HW/TL (heart weight/ Tibia length) ratio as a cardiac atrophy index of DOX or TUDCA treated mice.

Data represent the means ± SEM. (n = 5). P values were analyzed by one-way ANOVA test with Bonferroni post hoc test(c-e). ER indicates endoplasmic reticulum, TUDCA, Tauroursodeoxycholic Acid.


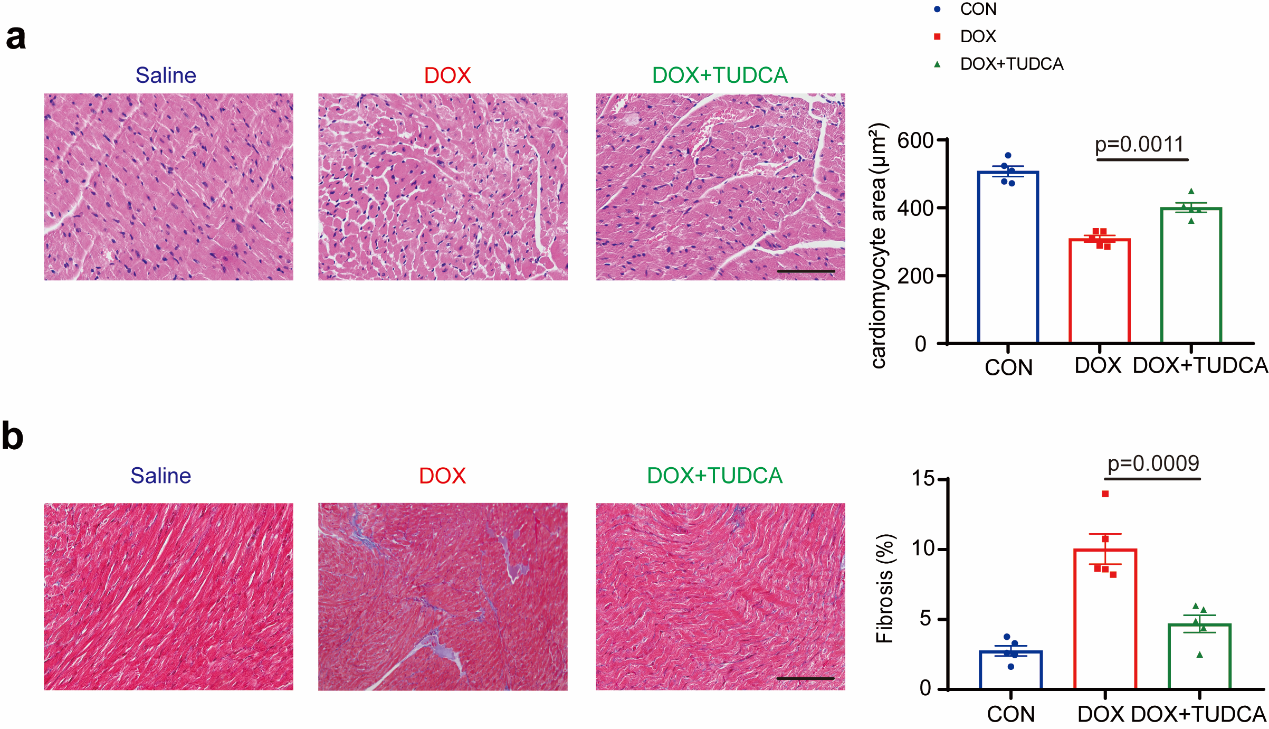


**Supplementary Fig. 15. Suppression ER stress attenuates the DOX-induced atrophy and fibrosis by chemical chaperone, TUDCA *in vivo***

(**a**) Representative images of H&E staining (left) and relative quantification (right) of cardiomyocyte area in heart sections. Scale bar, 50 µm.

(**b**) Representative images of Masson staining (left) and relative quantification (right) of collagen deposition in heart sections. Scale bar, 50 µm.

Data represent the means ± SEM. (n = 5). P values were analyzed by one-way ANOVA test with Bonferroni post hoc test(a and b). TUDCA indicates Tauroursodeoxycholic Acid.


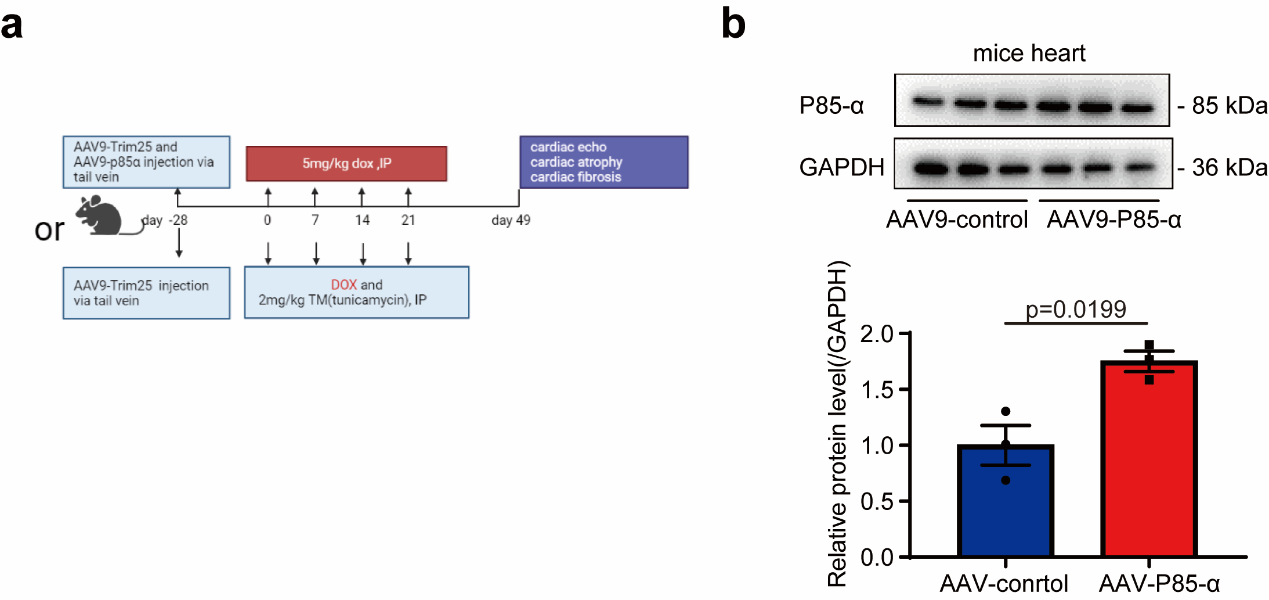


**Supplementary Fig. 16. Adeno-associated virus 9 expression efficiency on p85α in mouse heart**

(**a**) Schematic illustration showing chronic models of DOX cardiotoxicity, which were performed in mice to test the effect of overexpression of p85α or TM (Tunicamycin) the in heart.

(**b**) Representative western blot image showing p85α protein level in the heart of DOX-treated mice injected with AAV-Control or AAV- p85α.

(**c**) Representative mRNA level of p85α in heart of the mice.

Data represent the means ±SEM.(n = 3). P values were determined by unpaired 2-tailed Student t test.

Supplementary Table 1. Primary antibodies used in this study

| **Antibody** | **Company** | **Product**  **number** | **Dilution** | **Application** |
| --- | --- | --- | --- | --- |
| GAPDH | Proteintech | 60004-1-Ig | 1:10000 | WB |
| Bcl-2 | Cell Signaling Technology | 3498 | 1:1000 | WB |
| Bax | Cell Signaling Technology | 14796 | 1:1000 | WB |
| Cleaved Caspase-3 | Cell Signaling Technology | 9661 | 1:1000 | WB |
| Caspase-3 | Cell Signaling Technology | 9662 | 1:1000 | WB |
| TRIM25 | Cell Signaling Technology | 13773 | 1:1000 | WB |
| TRIM25 | Abcam | ab167154 | 1:100  1:500 | IP  IF |
| PI3 Kinase p85α | Cell Signaling Technology  Proteintech | 13666  60225-1-Ig | 1:1000 | WB  IP  IP |
| ATF-6 | Cell Signaling Technology | 65880 | 1:1000 | WB |
| BiP | Cell Signaling Technology | 3183 | 1:1000 | WB |
| CHOP | Cell Signaling Technology | 2895 | 1:1000 | WB |
| XBP-1s | Cell Signaling Technology | 40435 | 1:1000 | WB |
| P-IRE1 | Abcam | ab48187 | 1:1000 | WB |
| IRE1 | Abcam | ab37073 | 1:1000 | WB |
| P-JNK | Cell Signaling Technology | 4668 | 1:1000 | WB |
| JNK | Cell Signaling Technology | 9252 | 1:1000 | WB |
| Lamin A/C | Proteintech | 10298-1-AP | 1:1000 | WB |
| HA tag | Proteintech | 66006-1-Ig | 1:1000 | WB |
| Flag Tag | Sigma-Aldrich | F1804 | 1:1000 | WB |
| Anti-Flag Affinity Gel | Sigma-Aldrich | A4596 |  | IP |
| Protein G Agarose | Millopore | 16-266 |  | IP |

Supplementary Table 2. Primers used in quantitative RT-PCR

| **Gene** | **Forward primer(5’-3’)** | **Reverse primer(5’-3’)** |
| --- | --- | --- |
| Col1a1 | CA TGTTCAGCTTTGTGGACCT | GCAGCTGACTTCAGGGA TGT |
| Col3a1 | TCCCCTGGAA TCTGTGAA TC | TGAGTCGAA TTGGGGAGAA T |
| Ctgf | TGACCTGGAGGAAAACATTAA  GA | AGCCCTGTATGTCTTCACACTG |
| Nppa  (Anp) | CACAGATCTGATGGATTTCAAGA | CCTCA TCTTCTACCGGCA TC |
| Nppb  (Bnp) | GTCAGTCGTTTGGGCTGTAAC | AGACCCAGGCAGAGTCAGAA |
| Gapdh | AGGTCGGTGTGAACGGATTTG | TGTAGACCATGTAGTTGAGGTCA |

Supplementary Table 3

| Up regulated E3 ligases (GSE40289) | | |
| --- | --- | --- |
| \| BARD1 \| \| --- \| \| BRAP \| \| CBLL1 \| \| CNOT4 \| \| CRBN \| \| DCUN1D3 \| \| DTX2 \| \| E4F1 \| \| HERC6 \| \| ING1 \| \| MDM2 \| \| MYLIP \| \| NFX1 \| \| PELI1 \| \| PELI2 \| \| PPARG \| \| RANBP2 \| \| RASD2 \| \| RC3H1 \| \| RNF144B \| \| RNF146 \| \| RNF167 \| \| RNF181 \| \| RNF19B \| \| RNF2 \| \| RNF25 \| \| RNF31 \| \| RNF38 \| \| RNF4 \| \| RNF41 \| \| SYVN1 \| \| TOPORS \| \| TRAF5 \| \| TRAF6 \| \| TRIM21 \| \| TRIM25 \| \| TRIM26 \| \| TRIM27 \| \| TRIM39 \| | \| UBR4 \| \| --- \| \| VPS18 \| \| AHCTF1 \| \| BAZ1A \| \| ING3 \| \| INTS12 \| \| KDM2A \| \| NSD1 \| \| PHF8 \| \| RMND5A \| \| RNF39 \| \| SCAF11 \| \| SH3RF2 \| \| UPF1 \| \| WHSC1L1 \| \| AHR \| \| AMBRA1 \| \| ASB6 \| \| CORO7 \| \| DCAF13 \| \| EED \| \| ERCC8 \| \| FBXL14 \| \| FBXL3 \| \| FBXO30 \| \| FBXO33 \| \| FBXO7 \| \| FBXW7 \| \| FBXW8 \| \| GRWD1 \| \| MED8 \| \| NLE1 \| \| RAB40C \| \| RCBTB1 \| \| SOCS6 \| \| SPSB2 \| \| WDR5 \| \| WDR53 \| \| BTBD19 \| \| DCAF12L1 \| | \| ENC1 \| \| --- \| \| FBXW9 \| \| GEMIN5 \| \| KBTBD2 \| \| KBTBD4 \| \| KLHL35 \| \| MYNN \| \| PAK1IP1 \| \| PPWD1 \| \| PRPF4 \| \| PWP2 \| \| SSR3 \| \| TAF5L \| \| TCEB3 \| \| WDFY1 \| \| WDR3 \| \| WDR43 \| \| WDR48 \| \| WDR74 \| \| WDR75 \| \| WIPI2 \| \| ZBTB11 \| \| ZBTB16 \| \| ZBTB25 \| \| ZBTB38 \| \| ZBTB39 \| \| ZBTB41 \| \| ZBTB6 \| \| ZBTB9 \| |

| Down regulated E3 ligases (GSE40289) | | |
| --- | --- | --- |
| \| CBLC \| \| --- \| \| CBX4 \| \| CHFR \| \| CREBBP \| \| CUL9 \| \| DCUN1D2 \| \| HACE1 \| \| HECTD1 \| \| HERC4 \| \| MAP3K1 \| \| MGRN1 \| \| NARF \| \| NEURL3 \| \| NSMCE2 \| \| PARK2 \| \| PEX10 \| \| PJA2 \| \| PML \| \| PPIL2 \| \| RBCK1 \| \| RFFL \| \| RFWD3 \| \| RNF11 \| \| RNF121 \| \| RNF128 \| \| RNF13 \| \| RNF150 \| \| RNF170 \| \| RNF220 \| \| RNF24 \| \| RNF5 \| \| RNF6 \| \| RNF7 \| \| SMURF1 \| \| TRAF2 \| \| TRAF3 \| \| TRIM13 \| \| TRIM63 \| \| TRIM72 \| | \| TRIP12 \| \| --- \| \| UBE3C \| \| ZFP91 \| \| ZMIZ1 \| \| ZNRF1 \| \| CHD4 \| \| KDM5B \| \| NSD1 \| \| PDZRN4 \| \| PHF10 \| \| PHF2 \| \| PHF21A \| \| RNF157 \| \| RNFT2 \| \| TRIM16 \| \| TRIM55 \| \| ANAPC4 \| \| APC2 \| \| ASB12 \| \| ASB2 \| \| BRWD1 \| \| CDC27 \| \| CUL4A \| \| DCAF6 \| \| FBXL2 \| \| FBXL6 \| \| FBXO10 \| \| FBXO25 \| \| FBXO27 \| \| FBXO4 \| \| FBXO44 \| \| FBXW4 \| \| FZR1 \| \| PAFAH1B1 \| \| RBBP4 \| \| SPOP \| \| SPSB4 \| \| TAF5 \| \| TBL1X \| | \| WDR59 \| \| --- \| \| WDR82 \| \| ABTB2 \| \| ASB14 \| \| BTBD6 \| \| CORO6 \| \| EML1 \| \| EML2 \| \| GNB5 \| \| IBTK \| \| KBTBD12 \| \| KLHL30 \| \| KLHL31 \| \| MED16 \| \| NACC2 \| \| NBEAL1 \| \| POC1A \| \| RPTOR \| \| TLE6 \| \| TMEM183A \| \| WDFY3 \| \| WDR1 \| \| WDR37 \| \| WDR41 \| \| WDR72 \| \| WDR86 \| \| ZBTB20 \| \| ZBTB37 \| \| ZBTB7B \| |

Supplementary Table 4. Echocardiography values in the mice administrated with AAV9-GFP, AAV9-TRIM25-WT.

| Parameter | WT-Sham | WT-DOX | TRIM25-Sham | TRIM25-DOX |
| --- | --- | --- | --- | --- |
| Mice(N) | 10 | 10 | 8 | 10 |
| HR (bpm) | 533±20 | 518±13 | 522±6 | 524±16 |
| LVED;d (mm) | 3.50±0.08 | 3.59±0.05 | 3.55±0.05 | 3.49±0.16 |
| LVED;s (mm) | 2.26±0.07 | 2.61±0.07^**^ | 2.14±0.04 | 2.13±0.09^###^ |
| IVS;d (mm) | 0.71±0.02 | 0.61±0.06^**^ | 0.80±0.01 | 0.68±0.02 |
| IVS;s (mm) | 1.03±0.03 | 0.84±0.03^**^ | 1.26±0.05 | 1.04±0.03^##^ |
| LVPW;d (mm) | 0.70±0.03 | 0.64±0.02^***^ | 0.73±0.02 | 0.64±0.02 |
| LVPW;s (mm) | 0.97±0.04 | 0.80±0.02 | 1.07±0.03 | 0.92±0.01^#^ |

NOTE: Values are shown as mean ± SEM. **P<0.01 and ***P<0.001 vs. WT-Sham; ^#^P<0.05, ^##^P<0.01 and ^###^P<0.001 vs. WT-DOX by two-way repeated-measures ANOVA with Bonferroni’s post-hoc test.

Abbreviations: DOX, doxorubicin; HR, heart rate; LVEDd, left ventricular end-diastolic diameter; LVEDs, left ventricular end-systolic diameter; IVSd, interventricular septal thickness at end-diastole; IVSs, interventricular septal thickness at end-systole; LVPWd, left ventricular posterior wall thickness at end-diastole; LVPWs, left ventricular posterior wall thickness at end-systole.

Supplementary Table 5. Echocardiography values in the DOX-treated mice with TUDCA treatment.

| Parameter | WT-Sham | WT-DOX | TUDCA-DOX |
| --- | --- | --- | --- |
| Mice(N) | 5 | 5 | 5 |
| HR (bpm) | 510±14 | 483±28 | 464±22 |
| LVED;d (mm) | 3.52±0.05 | 3.47±0.08 | 3.30±0.17 |
| LVED;s (mm) | 2.23±0.07 | 2.53±0.09 | 2.50±0.10 |
| IVS;d (mm) | 0.75±0.03 | 0.60±0.06 | 0.61±0.04 |
| IVS;s (mm) | 1.23±0.08 | 0.80±0.03^***^ | 0.79±0.03 |
| LVPW;d (mm) | 0.64±0.05 | 0.57±0.03 | 0.72±0.02^#^ |
| LVPW;s (mm) | 0.96±0.09 | 0.76±0.05 | 0.91±0.07 |

NOTE: Values are shown as mean ± SEM. **P<0.01 and ***P<0.001 vs. WT-Sham; ^#^P<0.05, ^##^P<0.01 and ^###^P<0.001 vs. WT-DOX by two-way repeated-measures ANOVA with Bonferroni’s post-hoc test.

Abbreviations: DOX, doxorubicin; HR, heart rate; LVEDd, left ventricular end-diastolic diameter; LVEDs, left ventricular end-systolic diameter; IVSd, interventricular septal thickness at end-diastole; IVSs, interventricular septal thickness at end-systole; LVPWd, left ventricular posterior wall thickness at end-diastole; LVPWs, left ventricular posterior wall thickness at end-systole.

Supplementary Table 6. Echocardiography values in the mice administrated with AAV9-GFP, AAV9-TRIM25-WT and AAV9-p85α-WT.

| Parameter | WT-Sham | WT-DOX | TRIM25-DOX | TRIM25+p85α+DOX | TM+TRIM25+DOX |
| --- | --- | --- | --- | --- | --- |
| Mice(N) | 5 | 5 | 5 | 5 | 5 |
| HR (bpm) | 461±10 | 456±14 | 448±31 | 398±24 | 443±37 |
| LVED;d (mm) | 3.45±0.12 | 3.60±0.04 | 3.54±0.14 | 3.51±0.06 | 3.42±0.13 |
| LVED;s (mm) | 2.33±0.12 | 2.83±0.06^*^ | 2.50±0.10 | 3.51±0.06^@@@@^ | 3.42±0.13^@@@@^ |
| IVS;d (mm) | 0.69±0.03 | 0.56±0.06 | 0.66±0.02 | 0.56±0.03 | 0.55±0.04 |
| IVS;s (mm) | 1.10±0.04 | 0.81±0.03^***^ | 0.99±0.04 | 0.71±0.05^@@@^ | 0.76±0.03^@@^ |
| LVPW;d (mm) | 0.66±0.06 | 0.58±0.03 | 0.64±0.03 | 0.57±0.01 | 0.62±0.05 |
| LVPW;s (mm) | 0.85±0.05 | 0.81±0.05 | 0.90±0.01 | 0.76±0.02 | 0.88±0.04 |

NOTE: Values are shown as mean ± SEM. **P<0.01 and ***P<0.001 vs. WT-Sham; ^@^P<0.05, ^@@^P<0.01, ^@@@^P<0.001 and ^@@@@^P<0.001 vs. TRIM25-DOX by two-way repeated-measures ANOVA with Bonferroni’s post-hoc test.

Abbreviations: DOX, doxorubicin; HR, heart rate; LVEDd, left ventricular end-diastolic diameter; LVEDs, left ventricular end-systolic diameter; IVSd, interventricular septal thickness at end-diastole; IVSs, interventricular septal thickness at end-systole; LVPWd, left ventricular posterior wall thickness at end-diastole; LVPWs, left ventricular posterior wall thickness at end-systole.
