# Supplementary material for: Tripartite motif 25 ameliorates doxorubicin-induced cardiotoxicity by degrading p85α
Source: Cell Death Dis. 2022 Jul 23;13(7):643. doi: 10.1038/s41419-022-05100-4 (PMC9308790; doi:10.1038/s41419-022-05100-4)
Supplement: Supplementary file 3 — Original Data File [file 41419_2022_5100_MOESM3_ESM.pdf]

F1.f

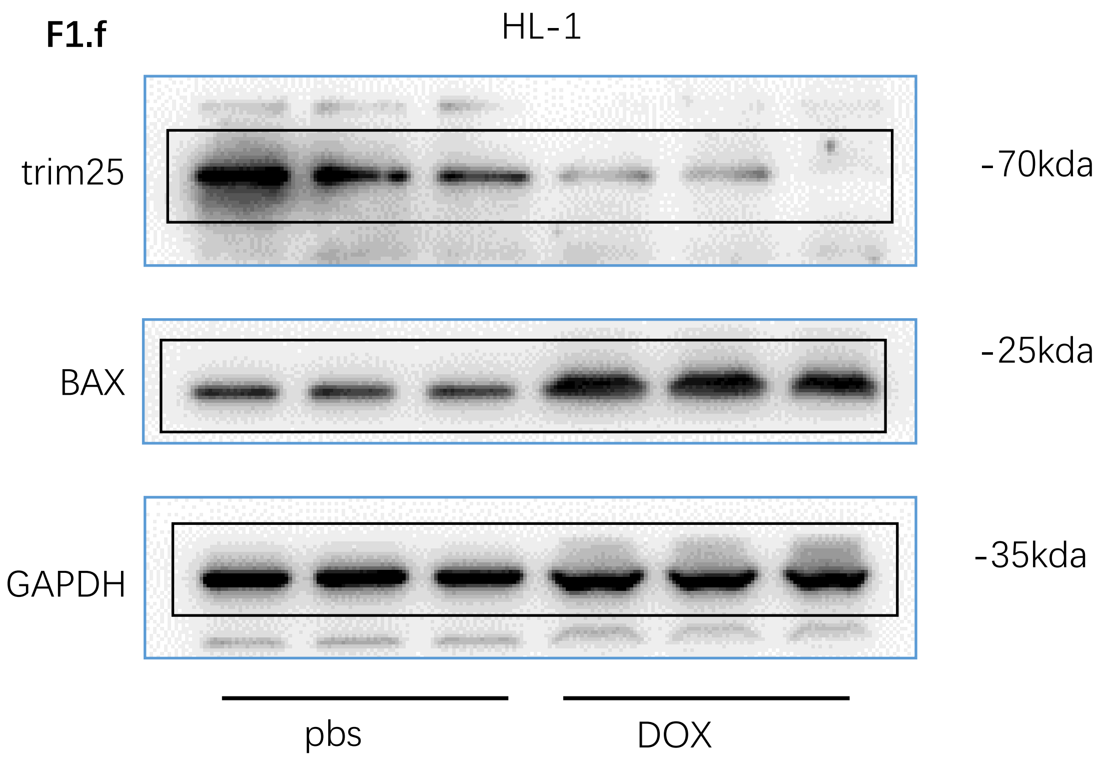
HL-1
trim25
-70kda
BAX
-25kda
GAPDH
-35kda
pbs
DOX

F1.g

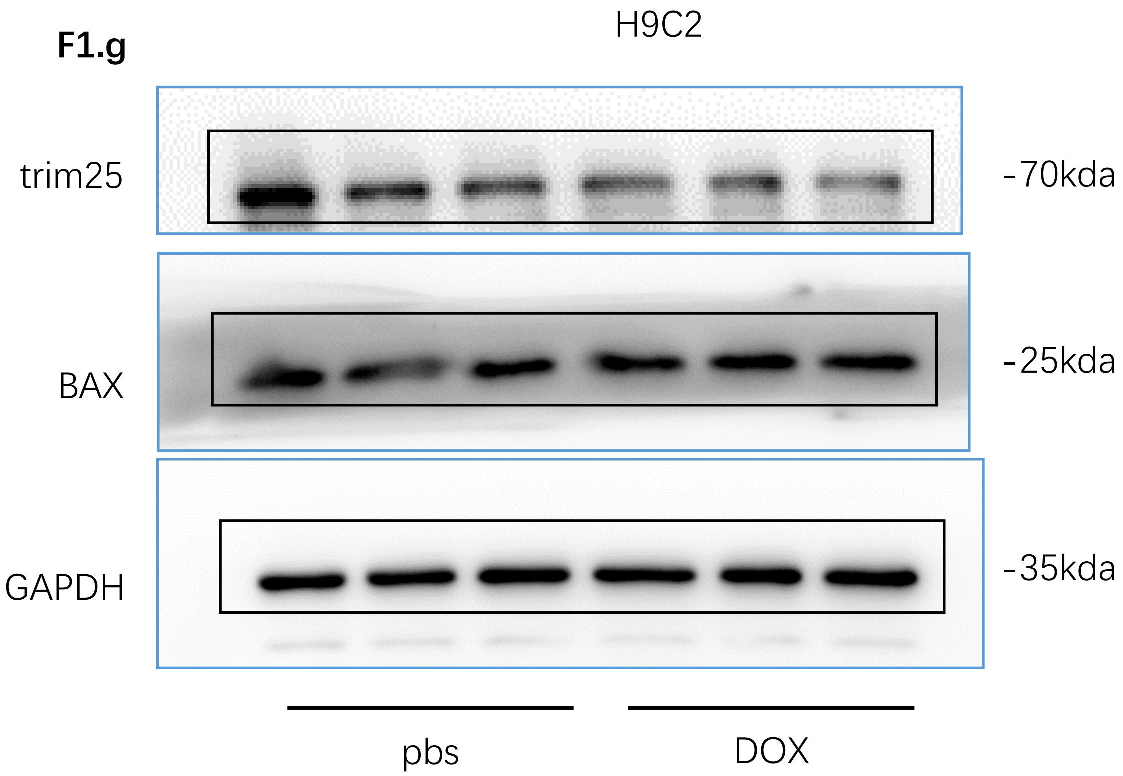
H9C2
trim25
-70kda
BAX
-25kda
GAPDH
-35kda
pbs
DOX

F1.i

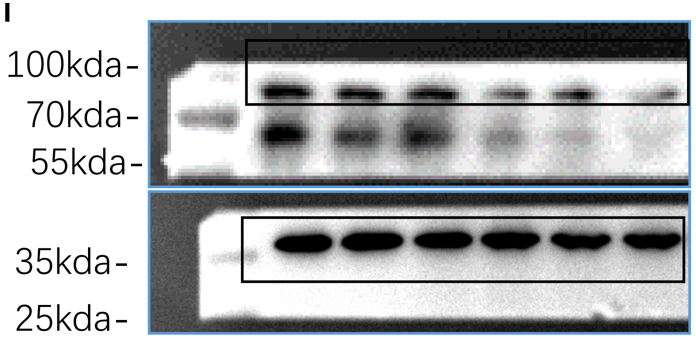
100kda-
70kda-
55kda-
35kda-
25kda-

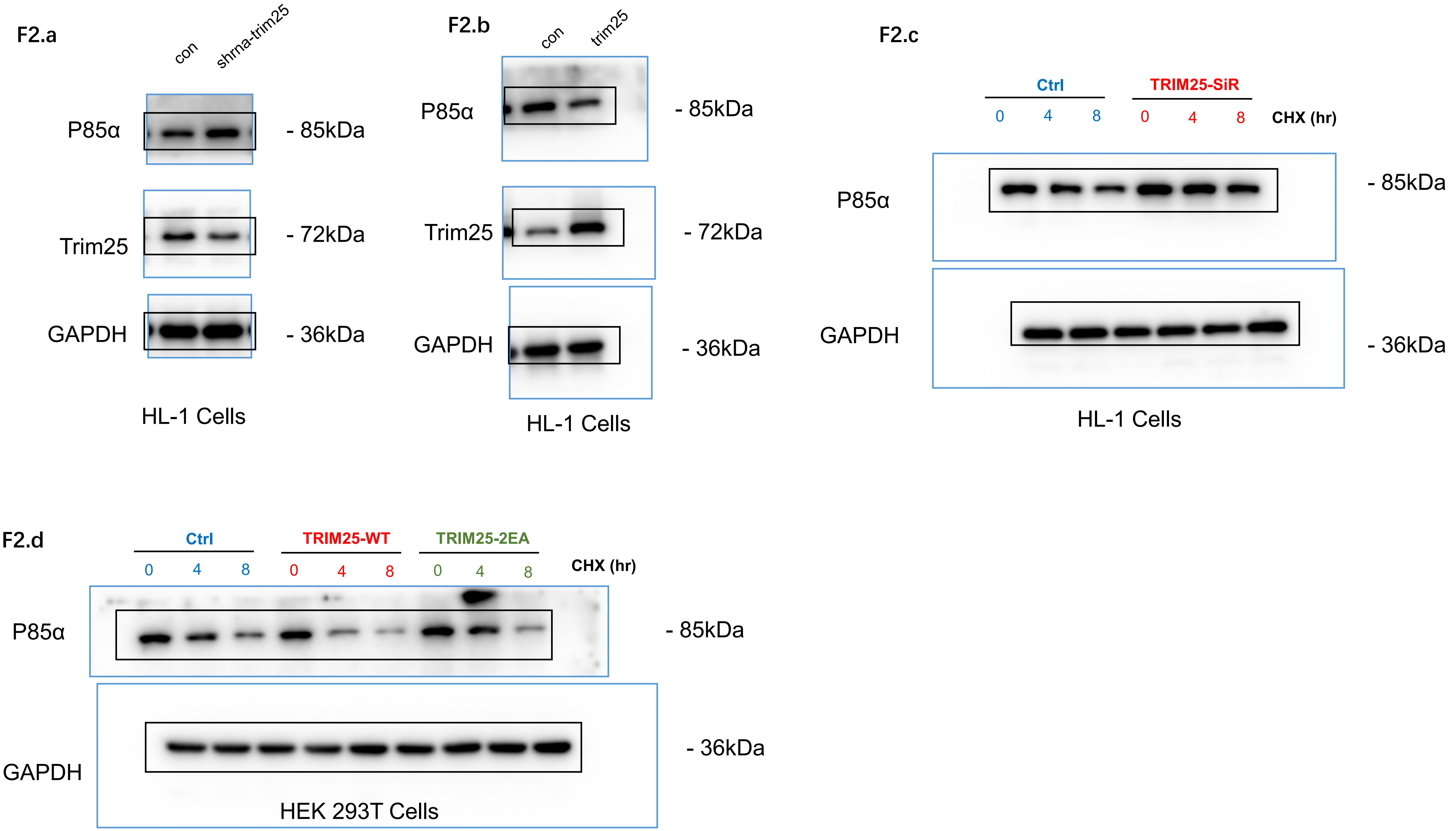

F2.a
con
shrna-trim25
P85α
- 85kDa
Trim25
- 72kDa
GAPDH
- 36kDa
HL-1 Cells
F2.b
con
trim25
P85α
- 85kDa
Trim25
- 72kDa
GAPDH
- 36kDa
HL-1 Cells
F2.c
Ctrl
TRIM25-SiR
0
4
8
0
4
8
CHX (hr)
P85α
- 85kDa
GAPDH
- 36kDa
HL-1 Cells
F2.d
Ctrl
TRIM25-WT
TRIM25-2EA
0
4
8
0
4
8
0
4
8
CHX (hr)
P85α
- 85kDa
GAPDH
- 36kDa
HEK 293T Cells

F2.g

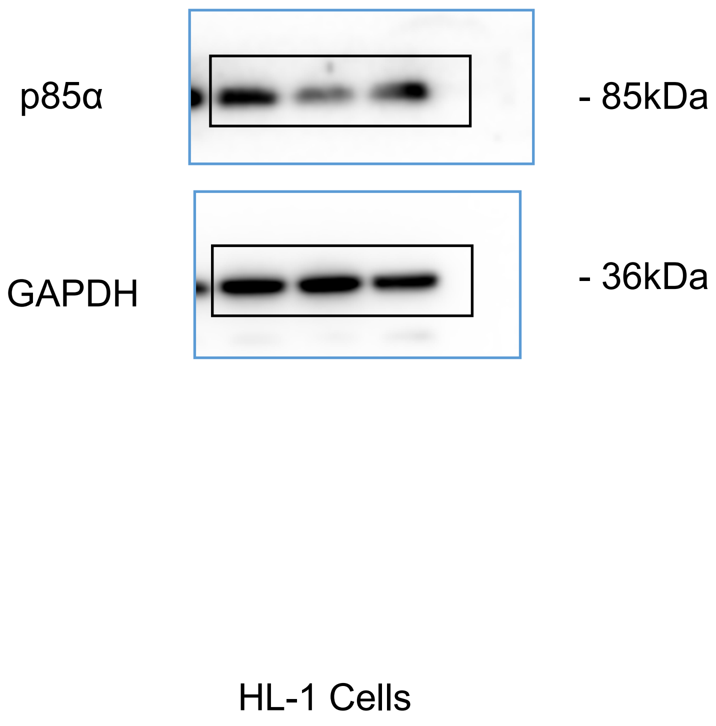
p85α
- 85kDa
GAPDH
- 36kDa
HL-1 Cells

F2.h

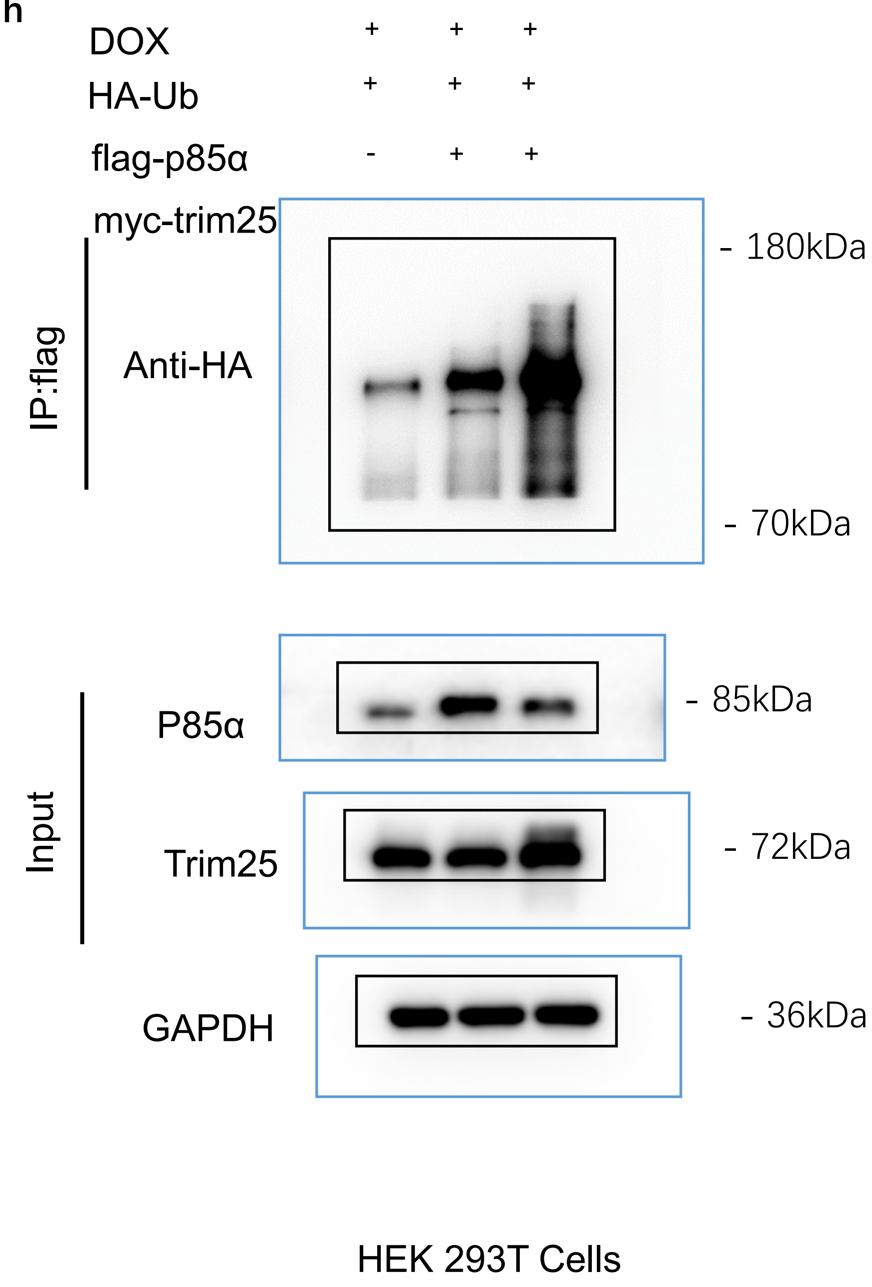
DOX + + +
HA-Ub + + +
flag-p85α - + +
myc-trim25
IP:flag
Anti-HA
- 180kDa
- 70kDa
Input
P85α
- 85kDa
Trim25
- 72kDa
GAPDH
- 36kDa
HEK 293T Cells

F2.e

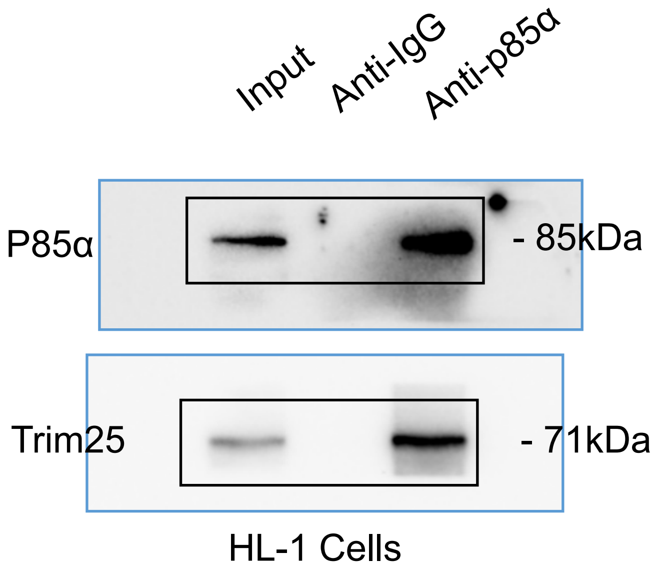

F2.f

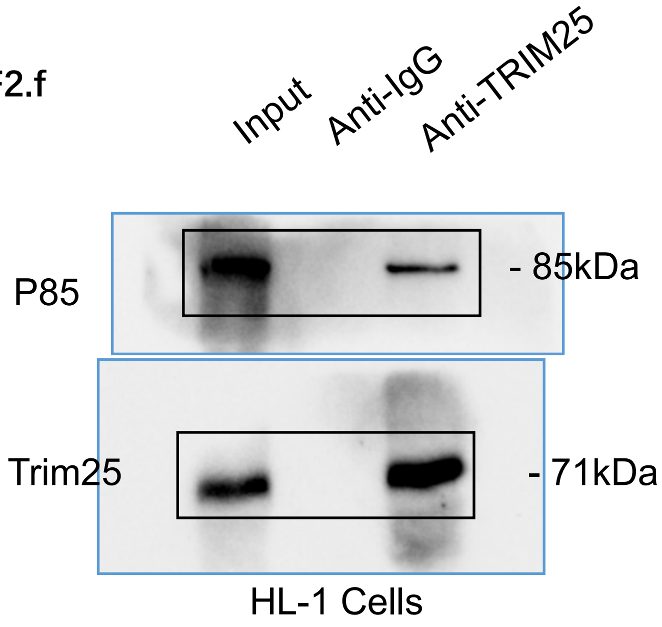

F3.a

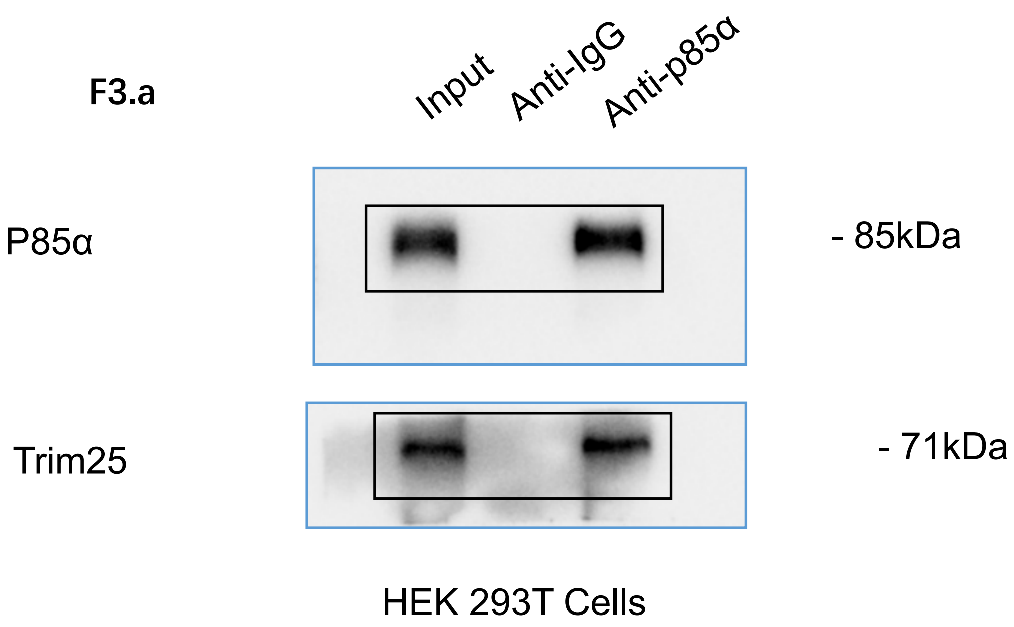

F3.b

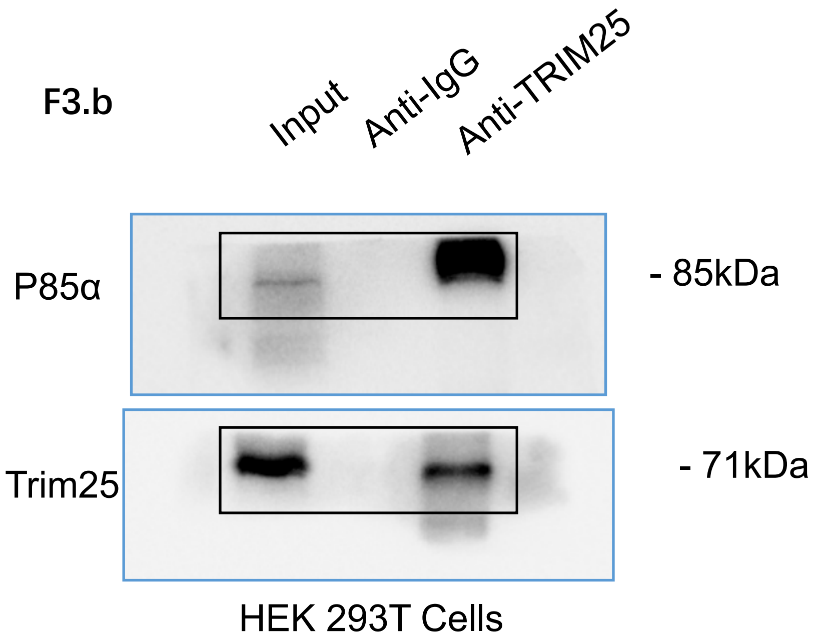

F3.c

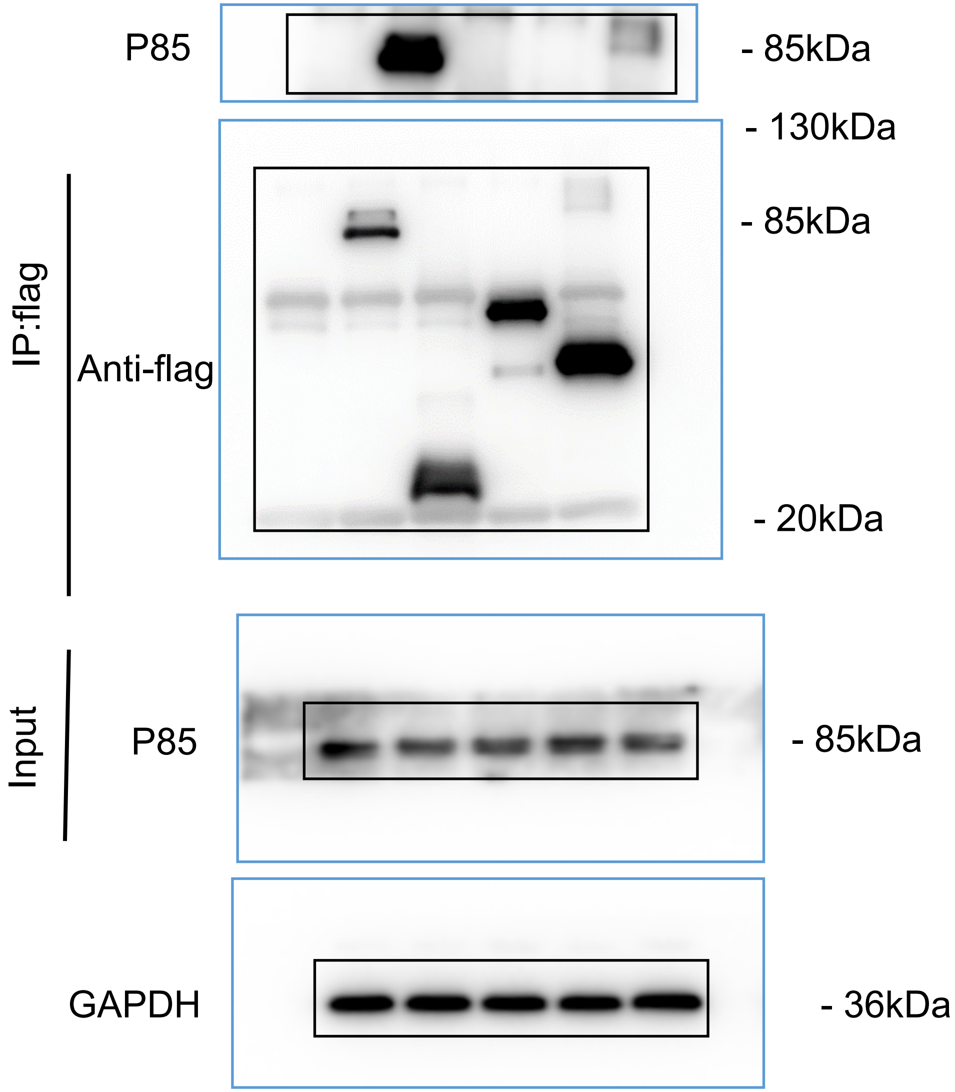
P85
- 85kDa
- 130kDa
- 85kDa
IP:flag
Anti-flag
- 20kDa
Input
P85
- 85kDa
GAPDH
- 36kDa

F3.d

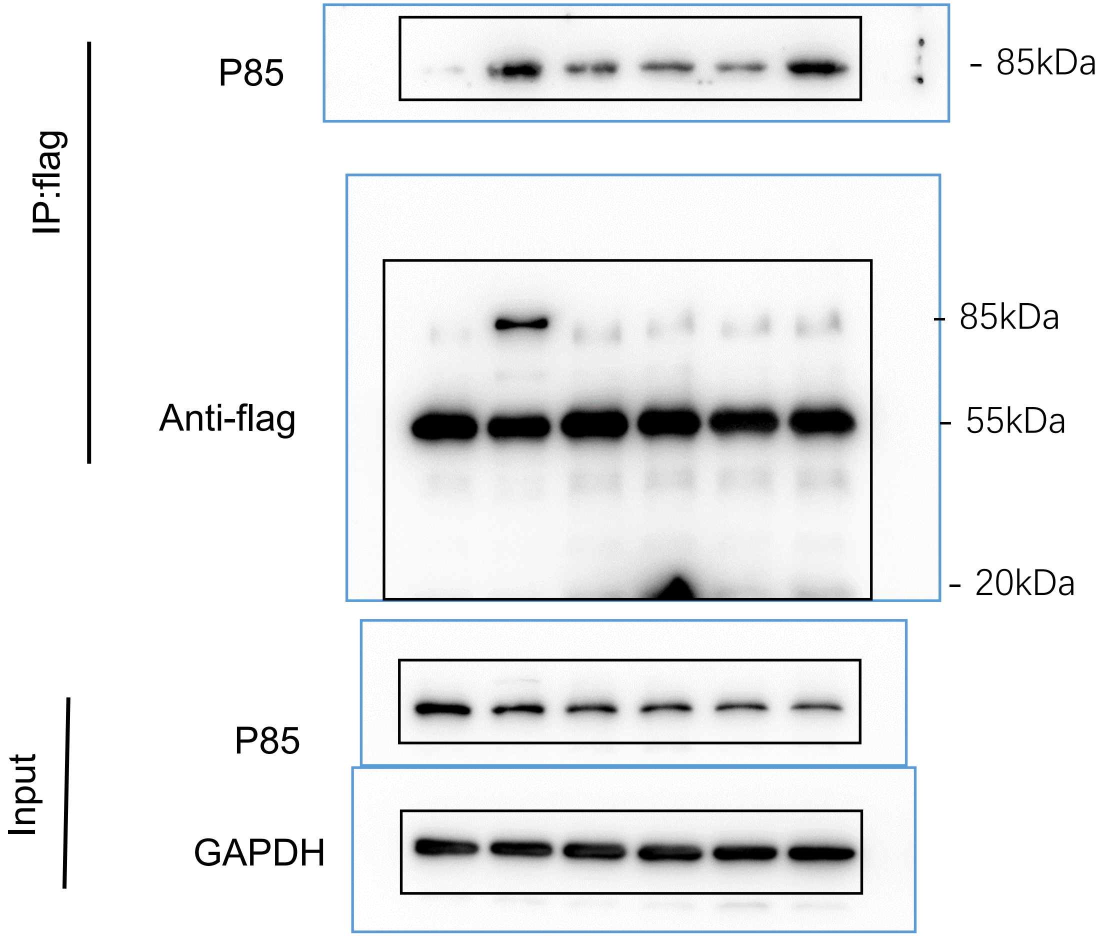
P85
- 85kDa
IP:flag
Anti-flag
- 85kDa
- 55kDa
- 20kDa
P85
Input
GAPDH

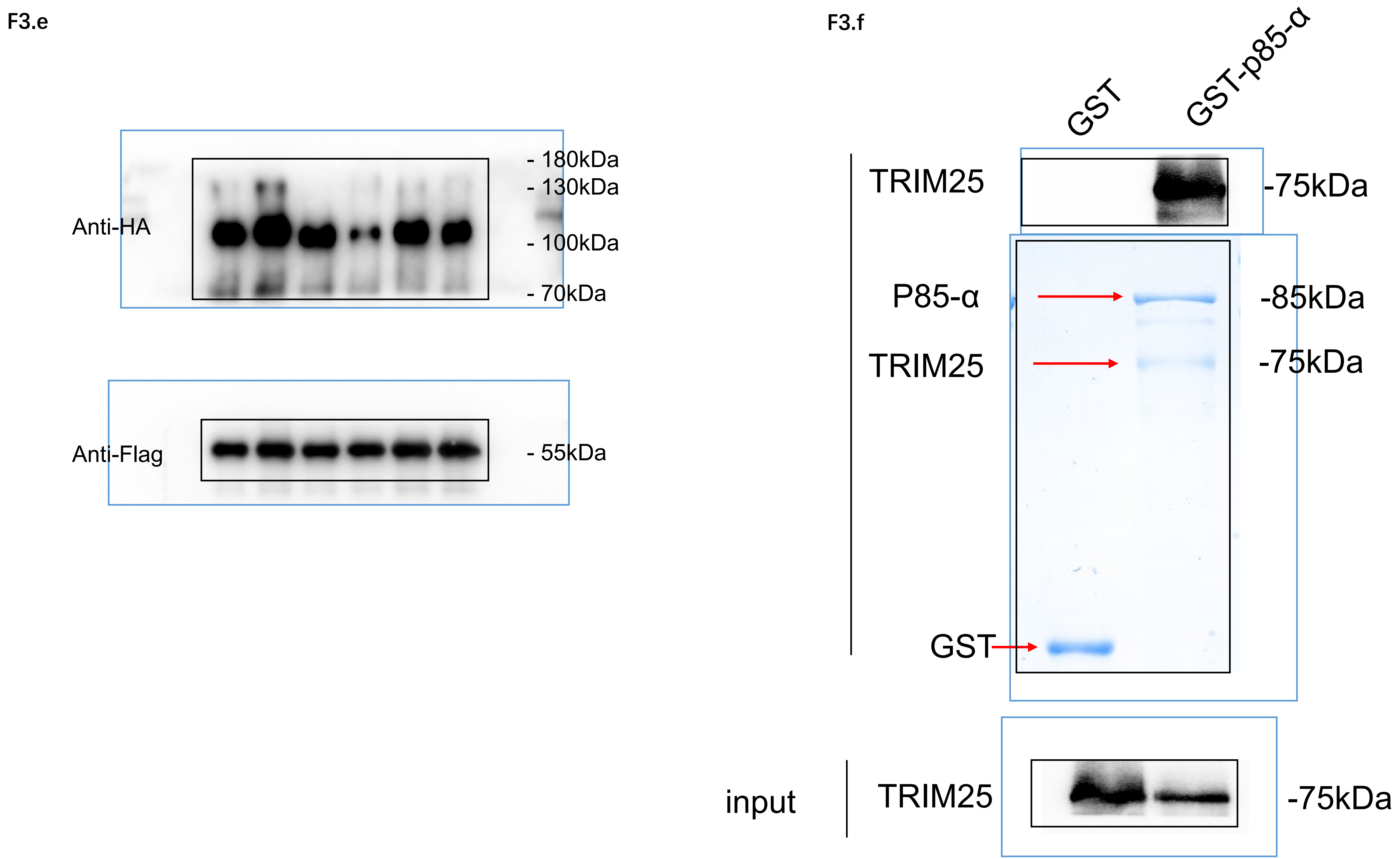
F3.e
Anti-HA
- 180kDa
- 130kDa
- 100kDa
- 70kDa
Anti-Flag
- 55kDa
F3.f
GST
GST-p85-α
TRIM25
-75kDa
P85-α
-85kDa
TRIM25
-75kDa
GST
input
TRIM25
-75kDa

F4.i

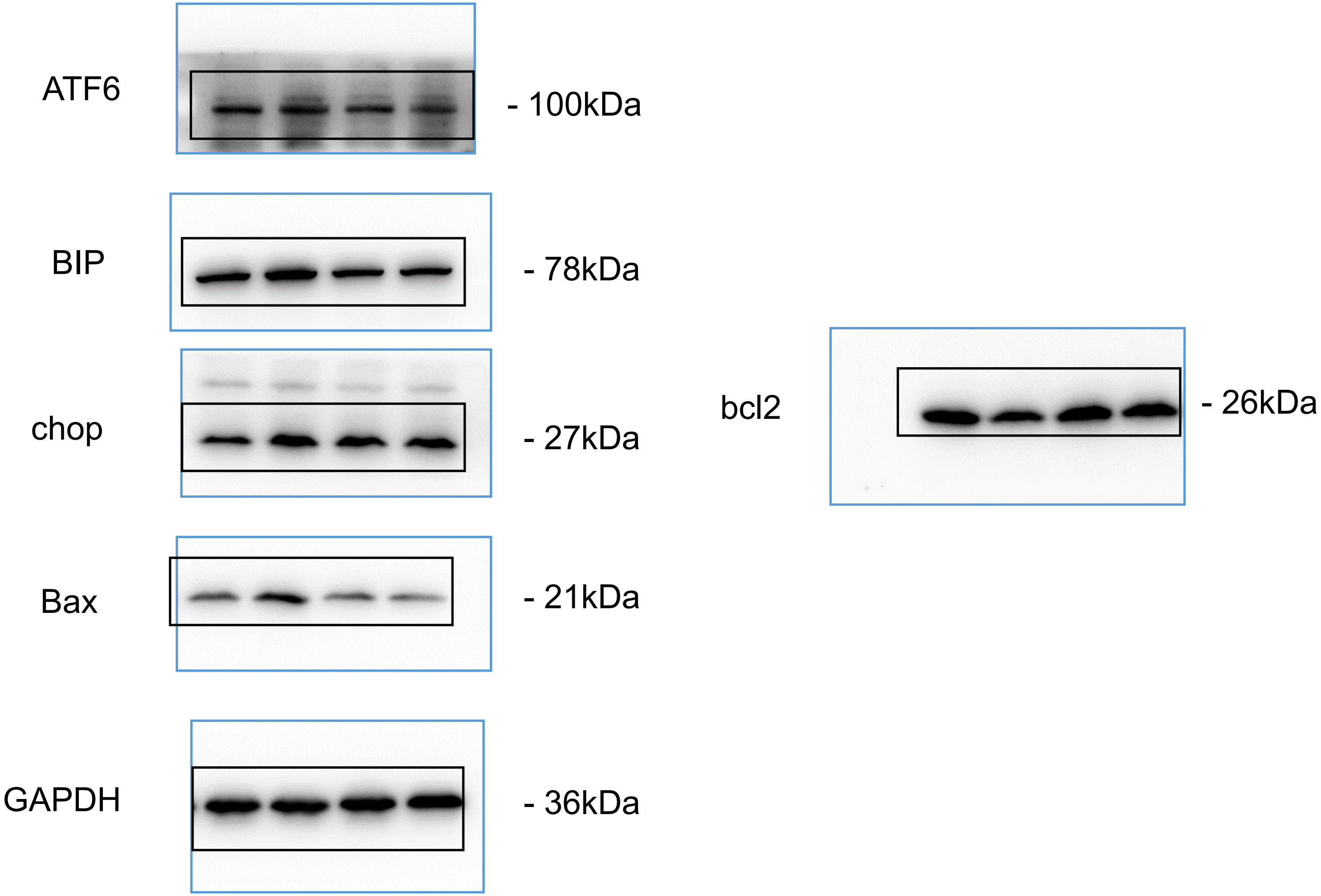
ATF6
- 100kDa
BIP
- 78kDa
chop
- 27kDa
Bax
- 21kDa
GAPDH
- 36kDa
bcl2
- 26kDa

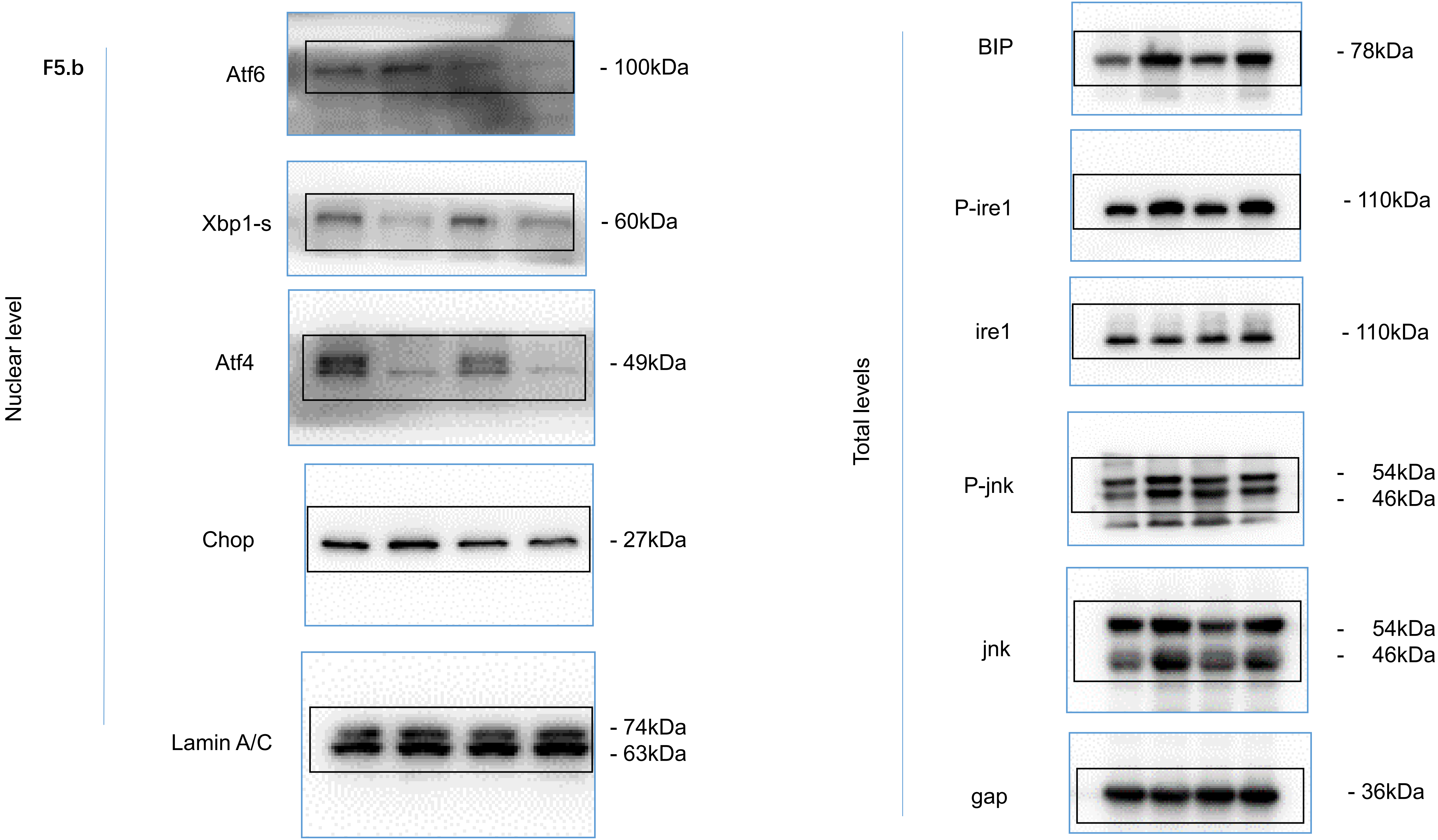
F5.b
Nuclear level
Atf6
- 100kDa
Xbp1-s
- 60kDa
Atf4
- 49kDa
Chop
- 27kDa
Lamin A/C
- 74kDa
- 63kDa
Total levels
BIP
- 78kDa
P-ire1
- 110kDa
ire1
- 110kDa
P-jnk
- 54kDa
- 46kDa
jnk
- 54kDa
- 46kDa
gap
- 36kDa

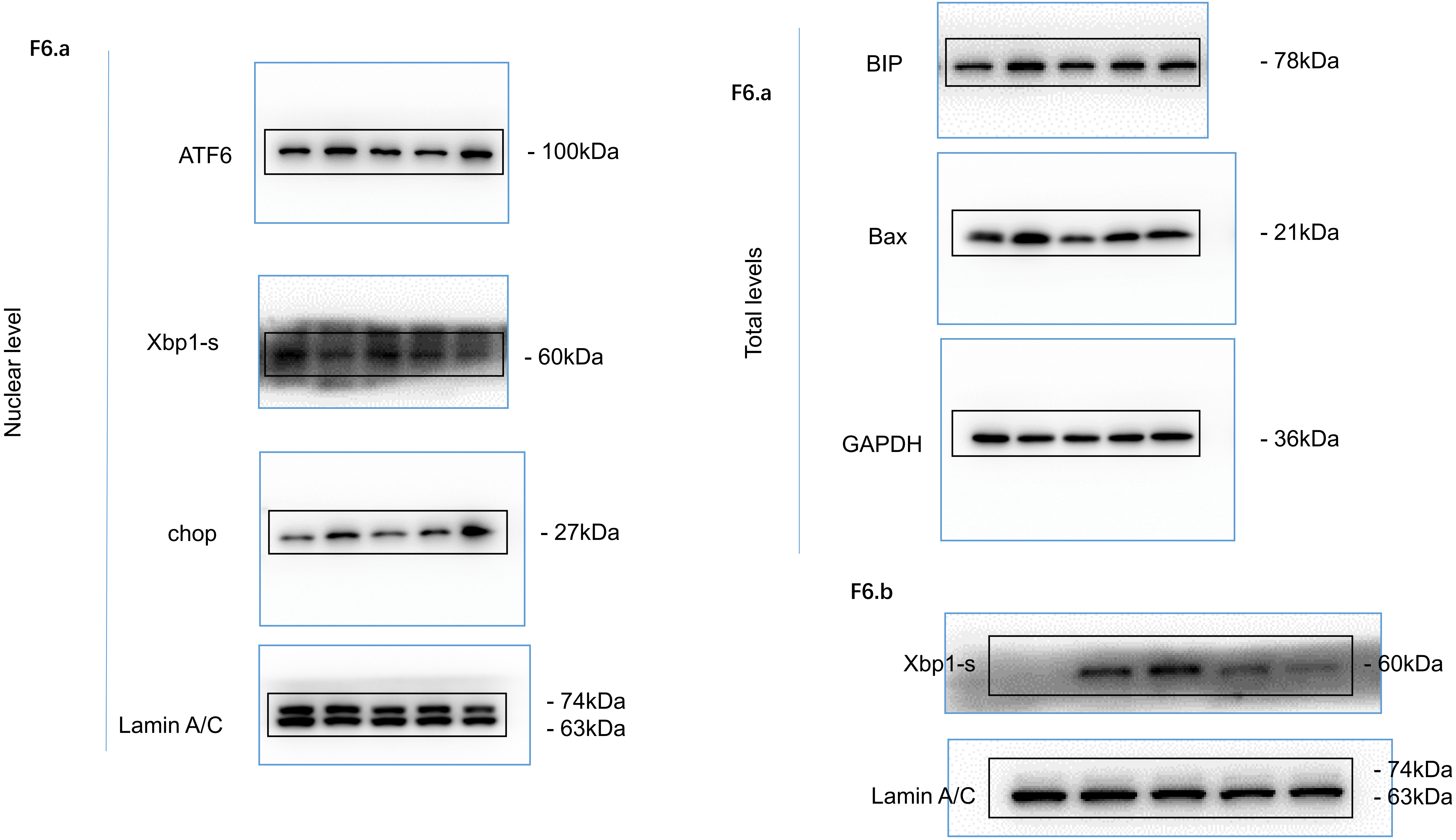
F6.a
Nuclear level
ATF6
- 100kDa
Xbp1-s
- 60kDa
chop
- 27kDa
Lamin A/C
- 74kDa
- 63kDa
F6.a
Total levels
BIP
- 78kDa
Bax
- 21kDa
GAPDH
- 36kDa
F6.b
Xbp1-s
- 60kDa
Lamin A/C
- 74kDa
- 63kDa

F6.c

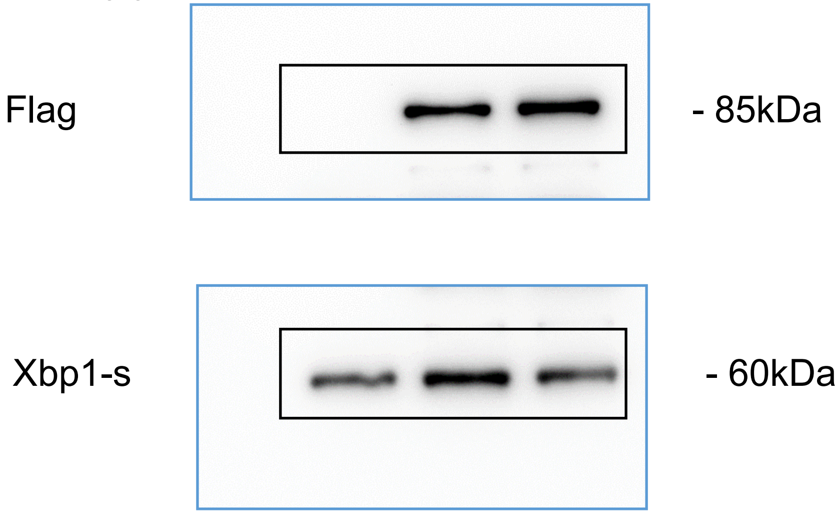
Flag
- 85kDa
Xbp1-s
- 60kDa

F6.d

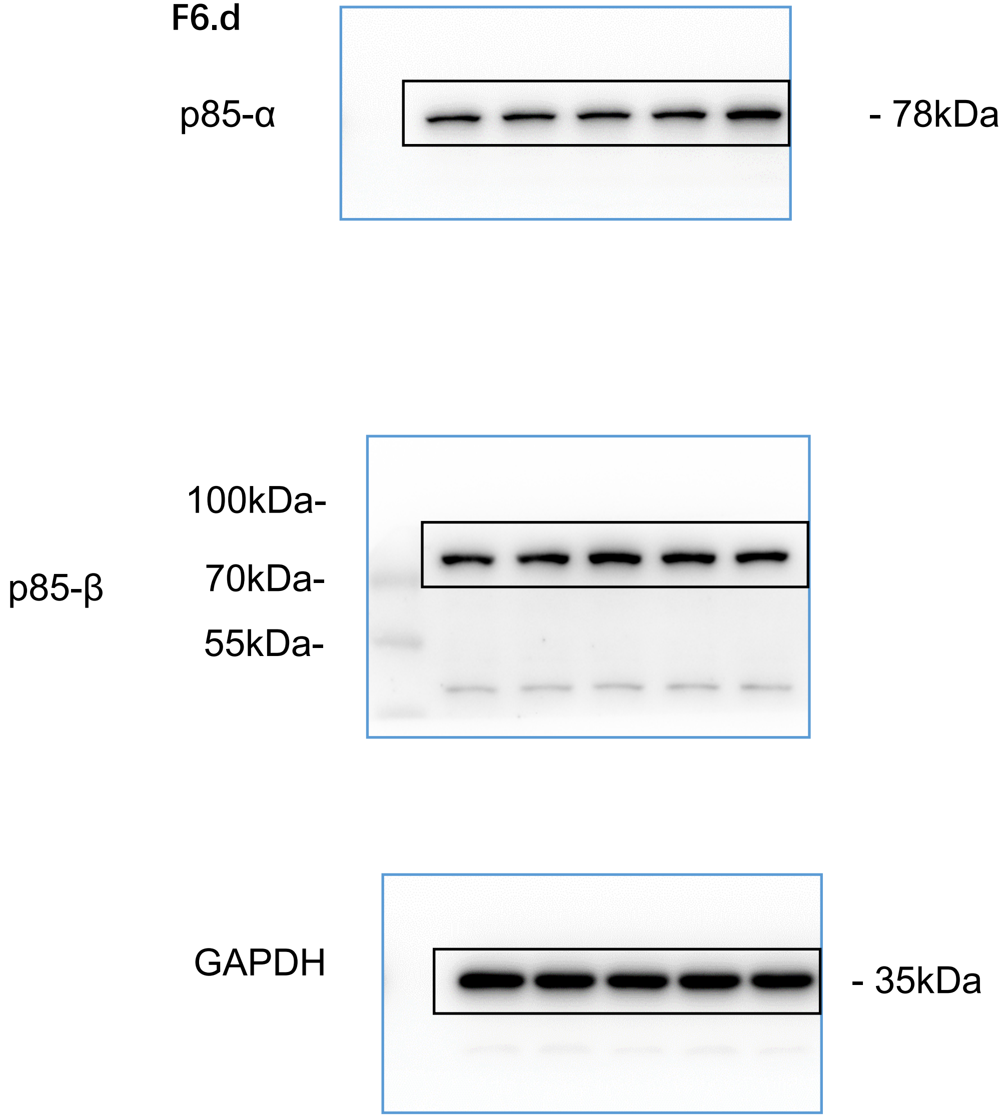
p85-α
- 78kDa
100kDa-
70kDa-
55kDa-
p85-β
GAPDH
- 35kDa

F7.g

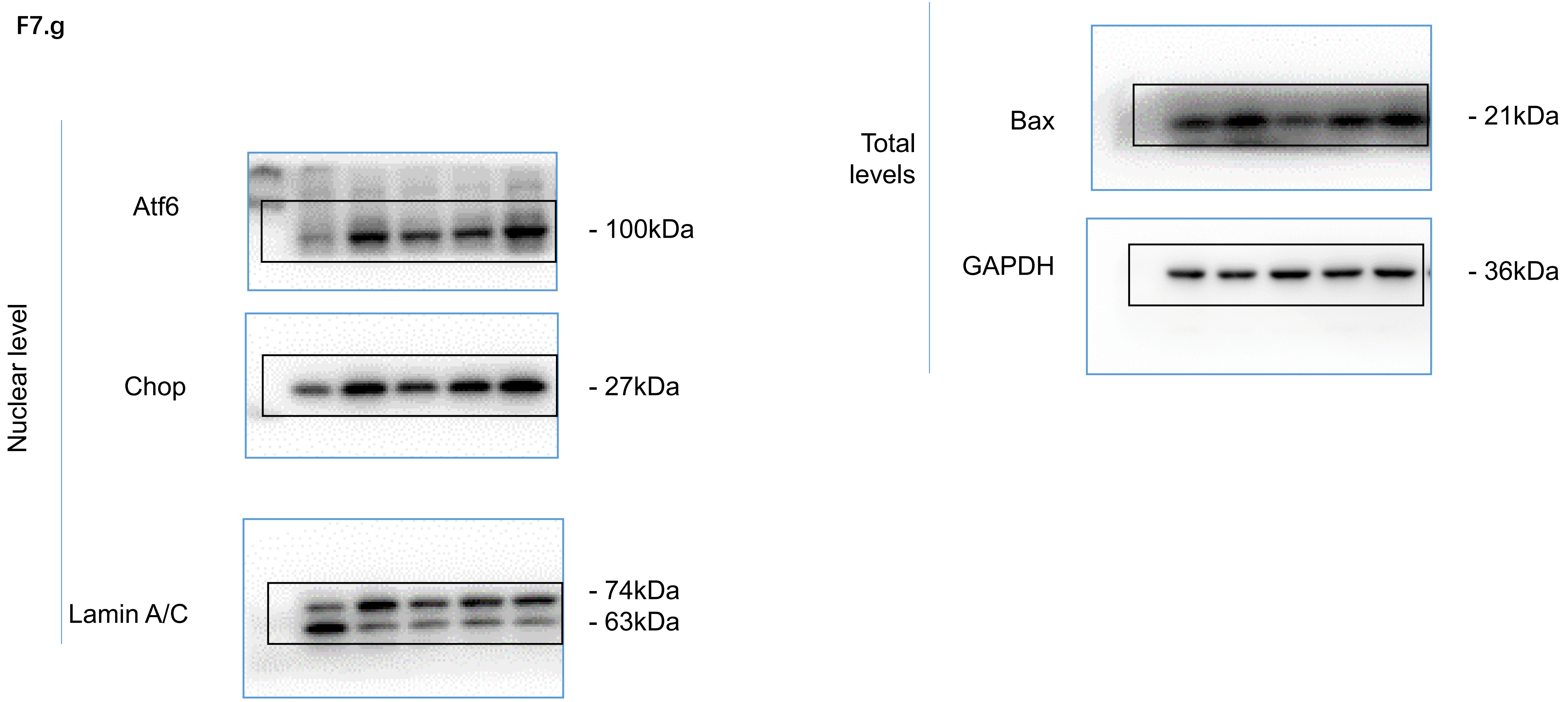
Nuclear level
Atf6
- 100kDa
Chop
- 27kDa
Lamin A/C
- 74kDa
- 63kDa
Total levels
Bax
- 21kDa
GAPDH
- 36kDa
