# Supplementary material for: Tripartite motif 25 ameliorates doxorubicin-induced cardiotoxicity by degrading p85α
Source: Cell Death Dis. 2022 Jul 23;13(7):643. doi: 10.1038/s41419-022-05100-4 (PMC9308790; doi:10.1038/s41419-022-05100-4)
Supplement: Supplementary file 4 — checklist [file 41419_2022_5100_MOESM4_ESM.pdf]

Reporting Summary

Springer Nature wishes to improve the reproducibility of the work that we publish. This checklist is used to ensure good reporting standards and to improve the reproducibility. Please respond completely to all questions relevant to your manuscript. For more information, please read the journal's Guide to Authors.

√ **Check here to confirm that the following information is available in the Material & Methods section:**

- **The exact sample size (*n*)** for each experimental group/condition, given as a number, not a range
- **A description of the sample collection** allowing the reader to understand whether the samples represent technical or biological replicates (including how many animals, litters, culture, etc.)

A statement of how many times the experiment shown was replicated in the laboratory

Definitions of statistical methods and measures: For small sample sizes (n<5) descriptive statistics are not appropriate, instead plot individual data points ○ Very common tests, such as *t*-test, simple χ^2 tests, Wilcoxon and Mann-Whitney tests, can be unambiguously identified by name only, but more complex techniques should be described in the methods section

○ Are tests one-sided or two-sided? ○ Are there adjustments for multiple comparisons? ○ **Statistical test results**, e.g., ***P* values** ○ Definition of **'center values'** as **median or mean**; ○ Definition of **error bars as s.d. or s.e.m. or c.i.**

Please ensure that the answers to the following questions are reported in the manuscript itself. We encourage you to include a specific subsection in the methods section for statistics, reagents and animal models. Below, provide the page number or section and paragraph number.

Statistics and general methods

Reported in section/paragraph or page #	
1. How was the sample size chosen to ensure adequate power to detect a pre-specified effect size? (Give section/paragraph or page #)	1.we chose the sample size by power calculations based on data. (Material and methods)
For animal studies, include a statement about sample size estimate even if no statistical methods were used.	Statement: Sample size was determined by power calculations based on data from preliminary experiments with an α level of 0.05 and power of 0.80, a minimum number of 5 mice per group was required. (Material and methods)
2. Describe inclusion/exclusion criteria if samples or animals were excluded from the analysis. Were the criteria pre-established? (Give section/paragraph or page #)	2. Every samples and animals were included in our study. (Material and methods)
3. If a method of randomization was used to determine how samples/animals were allocated to experimental groups and processed, describe it. (Give section/paragraph or page #)	3. a method of randomization was used in our study by Graphpad free online randomization tool. (Material and methods)
For animal studies, include a statement about randomization even if no randomization was used.	A simple and free online randomization tool was performed by Graphpad (https://www.graphpad.com/quickcalcs/randomize1.cfm). After the randomized allocation of animals to the treatments, animals, samples, and treatments are coded and recorded until the data are analyzed. (Material and methods)

4. If the investigator was blinded to the group allocation during the experiment and/or when assessing the outcome, state the extent of blinding. (Give section/paragraph or page #)	4.After the randomized allocation of animals to the treatments, animals, samples, and treatments are coded and recorded until the data are analyzed. (Material and methods)
For animal studies, include a statement about blinding even if no blinding was done.	After the randomized allocation of animals to the treatments, animals, samples, and treatments are coded and recorded until the data are analyzed. (Material and methods)
5. For every figure, are statistical tests justified as appropriate?	Yes
Do the data meet the assumptions of the tests (e.g., normal distribution)?	Yes
Is there an estimate of variation within each group of data?	Yes
Is the variance similar between the groups that are being statistically compared? (Give section/paragraph or page #)	Before using statistical analysis, Homogeneity of variance test was tested by bartlett testing. (Statistical analysis)

Reagents	Reported in section/paragraph or page #
6. Report the source of antibodies (vendor and catalog number)	The antibodies and catalog numbers in our study were listed in supplementary table 1. (Material and methods)
7. Identify the source of cell lines and report if they were recently authenticated (e.g., by STR profiling) and tested for mycoplasma contamination	The cell lines including mouse HL-1, H9C2, and HEK293T cells were all originally derived from from the Cell Bank of Chinese Academy of Sciences (Shanghai). The cell lines were all authenticated by Chinese Academy of Sciences. (Material and methods)

Animal Models	Reported in section/paragraph or page #
8. Report species, strain, sex and age of animals	Adult male C57BL/6 mice (8–10 weeks old, weighing 20-24g), purchased from Shanghai Laboratory Animal Center of Fudan university (Shanghai, China) were randomly assigned to each group for studies on Dox-induced cardiotoxicity. (Material and methods)
9. For experiments involving live vertebrates, include a statement of compliance with ethical regulations and identify the committee(s) approving the experiments.	Animals were housed in a specific pathogen-free facility according to the guidelines of the Care and Use of Laboratory Animals (published by the national institutes of health, NIH publication no.86-23, revised 1996). The experiments were approved by the animal care and use committee of Zhongshan Hospital Research Ethics. (Material and methods)

10. We recommend consulting the ARRIVE guidelines (*PLoS Biol.* **8**(6), e1000412,2010) to ensure that other relevant aspects of animal studies are adequately reported.

Human subjects

	Reported in section/paragraph or page #
11. Identify the committee(s) approving the study protocol.	human subjects were not involved in our study.
12. Include a statement confirming that informed consent was obtained from all subjects.	Not involved.
13. For publication of patient photos, include a statement confirming that consent to publish was obtained.	Not involved.
14. Report the clinical trial registration number (at ClinicalTrials.gov or equivalent).	Not involved.

15. For phase II and III randomized controlled trials, please refer to the CONSORT statement and submit the CONSORT checklist with your submission.

16. For tumor marker prognostic studies, we recommend that you follow the REMARK reporting guidelines.

Data deposition

	Reported in section/paragraph or page #
17. Provide accession codes for deposited data. Data deposition in a public repository is mandatory for:<br>a. Protein, DNA and RNA sequences<br>b. Macromolecular structures<br>c. Crystallographic data for small molecules<br>d. Microarray data	PXD032853 (https://www.iprox.cn/page/home.html),

Deposition is strongly recommended for many other datasets for which structured public repositories exist; more details on our data policy are available in the Guide to Authors. We encourage the provision of other source data in supplementary information or in unstructured repositories such as Figshare and Dryad. We encourage publication of Data Descriptors (see Scientific Data) to maximize data reuse.

18. If computer code was used to generate results that are central to the paper's conclusions, include a statement in the Methods section under "**Code availability"** to indicate whether and how the code can be accessed. Include version information as necessary and any restrictions on availability.	NA
